# Antimicrobial Susceptibility Pattern of *Helicobacter heilmannii* and *Helicobacter ailurogastricus* Isolates

**DOI:** 10.3390/microorganisms8060957

**Published:** 2020-06-25

**Authors:** Rita Matos, Chloë De Witte, Annemieke Smet, Helena Berlamont, Sofie De Bruyckere, Irina Amorim, Fátima Gärtner, Freddy Haesebrouck

**Affiliations:** 1 Instituto de Investigação e Inovação em Saúde da Universidade do Porto (i3S), 4200-135 Porto, Portugal; iamorim@ipatimup.pt (I.A.); fgartner@ipatimup.pt (F.G.); 2 Instituto de Ciências Biomédicas Abel Salazar, Universidade do Porto, 4050-313 Porto, Portugal; 3 Department of Pathology, Bacteriology and Avian Diseases, Faculty of Veterinary Medicine, 9820 Merelbeke, Belgium; helena.berlamont@ugent.be (H.B.); sofie.debruyckere@ugent.be (S.D.B.); Freddy.Haesebrouck@UGent.be (F.H.); 4 Translational Research in Immunology and Inflammation, Laboratory of Experimental Medicine and Pediatrics, Faculty of Medicine and Health Sciences, Antwerp University, B-2610 Antwerp, Belgium; annemieke.smet@uantwerpen.be

**Keywords:** *Helicobacter heilmannii*, *Helicobacter ailurogastricus*, antimicrobial susceptibility, zoonoses, gastric disease, resistance mechanisms

## Abstract

A combined agar and broth dilution method followed by qPCR was used to determine the antimicrobial susceptibility of feline *H. heilmannii* and *H. ailurogastricus* isolates. All *H. ailurogastricus* isolates showed a monomodal distribution of MICs for all the antimicrobial agents tested. For *H. heilmannii*, a bimodal distribution was observed for azithromycin, enrofloxacin, spectinomycin, and lincomycin. Single nucleotide polymorphisms (SNPs) were found in 50S ribosomal proteins L2 and L3 of the *H. heilmannii* isolate not belonging to the WT population for azithromycin, and in 30S ribosomal proteins S1, S7, and S12 of the isolate not belonging to the WT population for spectinomycin. The antimicrobial resistance mechanism to enrofloxacin and lincomycin remains unknown (2 and 1 *H. heilmannii* isolate(s), resp.). Furthermore, *H. heilmannii* isolates showed higher MICs for neomycin compared to *H. ailurogastricus* isolates which may be related to the presence of SNPs in several 30S and 50S ribosomal protein encoding genes and ribosomal RNA methyltransferase genes. This study shows that acquired resistance to azithromycin, spectinomycin, enrofloxacin, and lincomycin occasionally occurs in feline *H. heilmannii* isolates. As pets may constitute a source of infection for humans, this should be kept in mind when dealing with a human patient infected with *H. heilmannii*.

## 1. Introduction

*Helicobacter* species colonize the gastrointestinal tract of both humans and animals which may result in the development of gastrointestinal disorders such as inflammation, ulceration, and cancer. *Helicobacter pylori* is the best-studied and most prevalent *Helicobacter* species colonizing the stomach of human patients with gastric disorders [1,2,3,4,5,6]. Other gastric, spiral-shaped non-*H. pylori* helicobacters (NHPHs) have also been associated with the development of gastric disorders in humans [1,7,8,9]. So far, detected NHPHs in the human stomach are *H. suis*, *H. felis*, *H. bizzozeronii*, *H. salomonis*, and *H. heilmannii* [1,10,11,12,13,14]. *Helicobacter suis* naturally colonizes the stomach of pigs and non-human primates, while the others are associated with a wide range of canine and feline species. Living in close proximity as well as intense contact with infected animals are potential risk factors for humans to contract NHPH infection. The zoonotic potential of other gastric NHPHs, such as *H. cynogastricus*, *H. baculiformis*, *H. ailurogastricus*, *H. acinonychis*, *H. cetorum* and *H. mustelae*, is currently unknown.

*Helicobacter heilmannii* naturally colonizes the gastric mucosa of dogs and cats, with prevalence rates varying from 20% up to 100% [15,16,17]. Although infection has been associated with chronic active gastritis, peptic ulceration and chronic vomiting in dogs and cats, its pathogenic significance for these animal species is unclear and may be strain dependent [5,9,10,18,19,20,21,22,23,24]. In humans, *H. heilmannii* has been detected in 8–19% of gastric biopsies with histological evidence of NHPH infection [1,12,13,25]. In general, NHPH infections in human patients have been associated with gastritis, peptic ulcers, and mucosa-associated lymphoid tissue lymphoma (MALT) [6,23,25].

More recently, a new feline gastric *Helicobacter* species closely related to *H. heilmannii* has been described, namely, *H. ailurogastricus* [6]. Both species are phenotypically identical and cannot be distinguished by means of 16S rRNA and *ureAB* gene sequencing [6]. Although both species are morphologically very similar, *H. ailurogastricus* bacteria may be slightly smaller (3–5.5 µm length; 0.5–0.7 µm width) compared to *H. heilmannii* (3–6.5 µm length; 0.6–0.7 µm width) [6]. Both species present bipolar flagella and absence of periplasmic fibrils [6]. Biochemical analysis showed that the two species present urease activity, nitrate production, and both are able to hydrolyze indoxyl acetate, but only *H. ailurogastricus* shows alkaline phosphatase activity [6]. In vitro binding assays have shown that *H. ailurogastricus* has a lower capacity for binding to human- and murine-derived gastric epithelial cells than *H. heilmannii* which may explain why its virulence is lower than that of *H. heilmannii* [6]. Nevertheless, due to the fact of its recent discovery, the prevalence and pathogenic significance of *H. ailurogastricus* for animals and humans remains to be investigated.

Antimicrobial treatment of gastric NHPH infections in humans is based on clinical experience and mostly, treatment schemes applied for eradicating *H. pylori* are used [19]. *Helicobacter pylori* eradication treatment consists of 2–3 antibiotics (e.g., clarithromycin, amoxicillin, metronidazole, tetracycline, and/or levofloxacin) combined with an acid-suppressive drug (e.g., proton pump inhibitor-PPI or H2-receptor antagonists) [26]. However, not much is known on the antimicrobial susceptibility pattern of gastric NHPH species.

For some NHPH species, such as *H. suis*, *H. heilmannii*, and *H. ailurogastricus*, standard antimicrobial susceptibility assays are unsuitable since they only grow in an enriched biphasic medium with an acidic pH [6,8]. Therefore, Vermoote et al. [27] developed a combined agar and broth dilution method to analyze the antimicrobial susceptibility pattern of nine porcine *H. suis* isolates. More recently, the antimicrobial susceptibility of a larger collection of *H. suis* isolates obtained from pigs and non-human primates was investigated and acquired resistance to fluoroquinolones, spectinomycin, lincomycin, and tetracycline was shown [28]. So far, antimicrobial susceptibility testing has not been performed for *H. heilmannii* and *H. ailurogastricus*. These are very fastidious microorganisms which are difficult to cultivate. Therefore, only a limited number of isolates is available.

The aim of this study was to investigate the intrinsic susceptibility of *H. heilmannii* and *H. ailurogastricus* and the presence and mechanisms of acquired resistance in a collection of isolates obtained from the gastric mucosa of cats. This may eventually help to improve the management of infection with these bacteria in both humans and animals.

## 2. Materials and Methods

### 2.1. Minimal Inhibitory Concentration (MIC) Determination of *H. heilmannii* and *H. ailurogastricus* Isolates

Seven *H. heilmannii* (i.e., strains ASB1.4, ASB2.1, ASB3.2, ASB6.3, ASB14.1, ASB19, and ASB20) and six *H. ailurogastricus* (i.e., strains ASB7.1, ASB9.4, ASB11.2, ASB13.1, ASB21, and ASB23) isolates were included in this study. All strains were isolated by Smet et al. [17] and Joosten et al. [6] from the gastric mucosa of cats humanely euthanized at different animal shelters in Belgium or at the Faculty of Veterinary Medicine (Merelbeke, Belgium). Strains ASB2.1 and ASB3.2 were isolated from cats positive for feline immunodeficiency virus. No information on the health status of the other cats could be obtained, as they were “street cats” (*Felix vulgaris)* originating from different animal shelters. Whole genome sequence typing already demonstrated that all isolates were genetically different [6]. *Helicobacter heilmannii* and *H. ailurogastricus* isolates were cultivated according to the method described for *H. suis* by Berlamont et al. [28]. In brief, bacteria were grown using a biphasic medium consisting of Brucella agar (BD, Franklin Lakes, New Jersey, USA) supplemented with 20% inactivated fetal calf serum (Hyclone, ThermoFisher Scientific, USA), Vitox supplement (Oxoid, UK), and *Campylobacter* selective supplement (Skirrow, Oxoid, UK), with Brucella broth (Oxoid) added on top. Both agar and broth were supplemented with 0.05% HCl to obtain a pH of 5. Bacteria were incubated under microaerophilic conditions (85% N_2_, 10% CO_2_, 5% O_2_) at 37 °C. The concentration of viable bacteria was determined using a Neubauer improved counting chamber (Sigma–Aldrich).

The susceptibility of *H. heilmannii* and *H. ailurogastricus* to aminoglycosides (i.e., gentamicin and neomycin), β-lactam antibiotics (i.e., ampicillin and ceftiofur), fluoroquinolones (i.e., enrofloxacin and levofloxacin), lincomycin, macrolides (i.e., clarithromycin, azithromycin, and tylosin), metronidazole, rifampicin, spectinomycin, and tetracyclines (i.e., oxytetracycline and doxycycline) was determined using a combined agar and broth dilution method, according to the method described by Berlamont et al. [28]. The antimicrobial agents were purchased as standards powders from Sigma–Aldrich and were dissolved and diluted according to the Clinical & Laboratory Standards Institute (CLSI) guidelines. Serial two-fold dilutions of each antimicrobial agent were freshly prepared, with concentrations ranging from 0.003 to 128 µg/mL. *Helicobacter heilmannii* and *H. ailurogastricus* isolates were harvested and 150 µl of broth containing 5 × 10^7^ viable bacteria/mL was added to each corresponding well. Positive (i.e., wells containing agar, broth, and bacteria but without antimicrobial compound) and negative (i.e., wells without bacteria and antimicrobial compound) controls were included. All the concentrations and isolates were tested in duplicate. All the plates were incubated for 48 h under microaerophilic conditions at 37 °C. Broth containing *Helicobacter* spp. was collected from each well (i.e., approximately 100 µl) and bacterial DNA was extracted using PrepMan^®^ Ultra Sample Preparation Reagent (Applied Biosystems, CA, USA), according to the manufacturer’s instructions. Minimal inhibitory concentrations (MICs) were determined by software-assisted calculation of bacterial growth as determined by quantitative real-time PCR based on the *ureA* gene [21]. Similar to *H. suis*, the MIC was determined as the lowest concentration of the antimicrobial agent for which ΔCt was at least 1 Ct higher than ΔcCt (ΔCt = Ct after incubation – Ct before incubation of the antimicrobial agent exposed strains; ΔcCt = Ct after incubation – Ct before incubation of the controls; Ct = threshold cycle value) [20,25]. In other words, the MIC is the lowest concentration of the antimicrobial agent with at least 50% less bacterial growth [21,27,28].

The potency of the antimicrobials and the primers used for qPCR detection are listed in Tables S1 and S2, respectively.

### 2.2. Minimal Inhibitory Concentration Determination of Reference Strains

*Escherichia coli* ATCC 25922 and *Staphylococcus aureus* ATCC 29213 were used as reference strains. Both species were grown overnight and fresh suspensions with a density of 0.5 McFarland standard were prepared. The distribution of MIC values for both strains was determined using two different MIC assays as described by Berlamont et al. [28]. In brief, antimicrobial susceptibility patterns of both reference strains were determined using the combined agar and broth dilution method with a pH of 5 (assay (1)) as well as the broth microdilution method according to the CLSI standards at pH 7 (assay (2)). For assay (1), *E. coli* and *S. aureus* were cultured in the biphasic conditions at pH 5, as described for *H. heilmannii* and *H. ailurogastricus*. For assay (2), unsupplemented Mueller–Hinton broth at pH 7 was used according to the CLSI guidelines. Additionally, positive (i.e., wells containing agar, broth, and bacteria but free of the tested antimicrobials) and negative (i.e., wells containing agar and broth free of the tested antimicrobials and reference bacteria) controls were included. All samples were tested in duplicate. Plates were incubated for 16–20 h under aerobic conditions at 37 °C. The presence of bacterial growth was evaluated by the presence of turbidity, and the value was defined as the lowest concentration for which turbidity was visually absent.

### 2.3. *Helicobacter* Resistance Mechanisms to Antimicrobial Agents

Genome sequences of all tested *H. heilmannii* and *H. ailurogastricus* isolates are available from the ftp NCBI database (Table S3). As described by Berlamont et al. [28], multiple tools were used to screen these genomes for the presence of acquired antimicrobial resistance genes, namely: ABRicate [29] Resfinder [30] ARG-ANNOT [31], The Comprehensive Antibiotic Resistance Database (CARD) [32], NCBI Bacterial Antimicrobial Resistance Reference Gene Databases [33], EcOH [34], PlasmidFinder [35], and Virulence Factors Database (VFDB) [36]. In addition, the presence of point mutations in specific gene sequences from *H. heilmannii* and *H. ailurogastricus* isolates (not) belonging to the wild-type population [37] were screened manually using Clustal Omega [38], PredictSNP [39], and ConSurf [28,40]. Single nucleotide polymorphisms (SNPs) were only considered relevant if they were detected in all isolates not belonging to the wild-type population for a certain antimicrobial agent, while absent in all isolates belonging to the wild-type population for that antimicrobial agent.

### 2.4. Ethical Statement

Procedures for strain isolation were approved by the Ethical Committee of the Faculty of Veterinary Medicine, Ghent University, Merelbeke, Belgium (approval number EC2011/090).

### 2.5. Statistical Analysis

Average and standard deviation were calculated for each antimicrobial agent for all the *H. heilmannii* and *H. ailurogastricus* strains belonging to the wild-type population The MIC pattern of *H. heilmannii* isolates was compared to the *H. ailurogastricus* isolates, using a two-way ANOVA test (Sidak’s test). Differences were considered statistically significant at a *p*-value of 0.05. GraphPad Prism 7 software was used for the analysis.

## 3. Results

### 3.1. Antimicrobial Activity at Different pH

Two different MIC assays were performed to determine the impact of culture and pH conditions. When using the combined agar and broth dilution method at pH 5 on the *E. coli* and *S. aureus* reference strains, the MIC values of spectinomycin were two times, 2-fold dilutions; for azithromycin, clarithromycin, enrofloxacin, and lincomycin three times, 2-fold dilutions; for tylosin and levofloxacin four times, 2-fold dilutions; and for gentamicin and neomycin six times, 2-fold dilutions higher than the highest value of the acceptable quality ranges of the CLSI standards. On the other hand, MIC values of ceftiofur and ampicillin were one, 2-fold dilution lower than the lowest value of the acceptable quality ranges of the CLSI standards when using the combined agar and broth dilution method at pH 5 [41]. Minimal inhibitory concentration (MIC) endpoints of doxycycline, tetracycline, metronidazole, and rifampicin fell within the acceptable quality ranges when using the combined agar and broth method at pH 5.

When using the broth microdilution method according to the CLSI guidelines, at pH 7, on both reference strains, the MIC endpoints of all antimicrobial agents fell within the acceptable quality control ranges [41].

### 3.2. Minimal Inhibitory Concentration Pattern of *H. heilmannii* and *H. ailurogastricus*

All positive control plates showed bacterial growth and all negative controls did not show bacterial growth, indicating that all experiment conditions were performed correctly.

**Figure 1:**
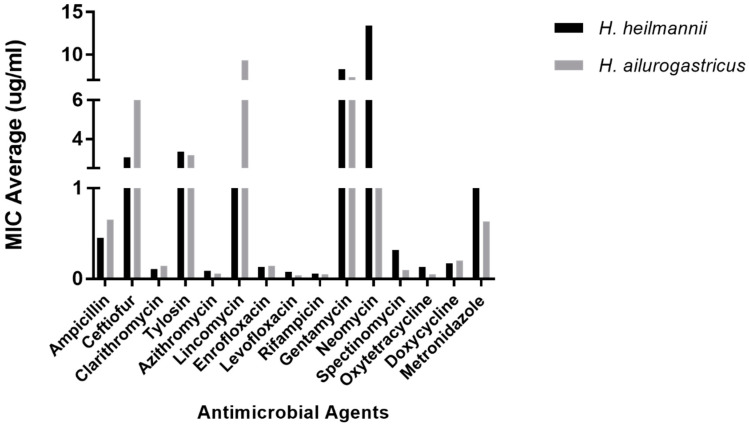
Graphical representation of distribution of MIC values of *H. heilmannii* and *H. ailurogastricus* isolates to the different tested antimicrobial agents. Statistical analysis was performed using the GraphPad software, but no significant differences were found between both species.

The distribution of MIC values for all *H. heilmannii* and *H. ailurogastricus* isolates is shown in Table 1 and Figure 1. Average and standard deviation of *H. heilmannii* and *H. ailurogastricus* isolates for each antimicrobial agent are presented in Table S4. All *H. ailurogastricus* isolates showed a monomodal distribution for all the antimicrobial agents tested. For the *H. heilmannii* isolates, a monomodal distribution of MICs was observed for azithromycin, β-lactam antibiotics (i.e., ampicillin and ceftiofur), clarithromycin, gentamicin, neomycin, nitroimidazole antibiotics (i.e., metronidazole), levofloxacin, rifamycins (i.e., rifampicin), tetracyclines (i.e., oxytetracycline and doxycycline) and tylosin. Interestingly, a bimodal distribution was seen for azithromycin (i.e., strain ASB19), enrofloxacin (i.e., strains ASB14.1 and ASB20), lincomycin (i.e., strain ASB6.3), and spectinomycin (i.e., strain ASB1.4). The presence of a bimodal distribution indicates that the isolates showing a higher MIC range did not belong to the wild-type population for these antimicrobial agents.

Differences in the distribution of MIC values between *H. heilmannii* and *H. ailurogastricus* were observed for lincomycin and neomycin. For lincomycin, higher MIC values were observed for *H. ailurogastricus* isolates compared to *H.* heilmannii. In more detail, for *H. ailurogastricus*, the MIC values for lincomycin varied between 4–16 µg/mL (9.33 ± 5.47 µg/mL), while for *H. heilmannii*, the MIC values varied between 0.5–2 µg/mL (1.25 ± 0.61 µg/mL) with the exception of *H. heilmannii* ASB6.3 showing a high MIC value of 32 µg/mL (Table 1 and Table S4). Compared to *H. heilmannii* isolates, *H. ailurogastricus* isolates showed lower MIC values. More specifically, for *H. heilmannii*, the MIC values for neomycin varied between 2–32 µg/mL (13.43 ± 10.11 µg/mL), while for *H. ailurogastricus* the MIC values varied between 0.5–2 µg/mL (1.33 ± 0.75 µg/mL) (Table 1 and Table S4). For isolates belonging to the wild-type population, no significant differences were detected between both species for all the antimicrobial agents tested (Figure 1 and Table S4).

### 3.3. Antimicrobial Resistance Mechanisms Presented by *Helicobacter* species

No known acquired antimicrobial resistance genes were detected in the *H. heilmannii* and *H. ailurogastricus* isolates. Although point mutations in the 23S rRNA genes were associated with resistance of *H. pylori* and other pathogens to macrolides, none were found in the *H. heilmannii* isolate ASB19 not belonging to the wild-type population for azithromycin.

Specific point mutations in the *gyrA* and *gyrB* genes were associated with resistance of *H. pylori* and *Enterobacteriaceae* to fluoroquinolones. Nevertheless, no known point mutations were detected in *H. heilmannii* isolates ASB14.1 and ASB20 not belonging to the wild-type population for enrofloxacin.

Single nucleotide polymorphisms were found in two 50S ribosomal protein genes of the *H. heilmannii* isolate ASB19 not belonging to the wild-type population for azithromycin (i.e., MIC value of 4 µg/mL) which are shown in Table S5. For example, SNPs were found in the 50S ribosomal protein L3 (*RplC*) at codon 259 (CCT (proline) -> TCT (serine)) and at codon 277 (CGC (arginine) -> TGC (cysteine)). Using BLAST, various amino acids were present at these positions in other bacterial species and the ConSurf Server gave conservation scores below five. Nevertheless, cysteine was never present at codon 277 in other bacterial species. None of the PredictSNP tools showed that the amino acid substitution at codon 259 may affect *RplC* activity. Three PredictSNP tools—MAPP, PhD-SNP, and PolyPhen-1—indicated that the amino acid substitution at codon 277 may affect *RplC* activity with an average accuracy of 56%, while the other tools indicated a neutral effect with an average accuracy of 60%. Using the I-Mutant 3.0 tool, the amino acid substitution at both codon 259 and 277 were predicted to decrease protein stability (DDG: −1.31, RI: 8; DDG: −0.64, RI:0, resp.). Analysis of the SNP present in 50S ribosomal protein L2 gene (*RplB*) indicated that this amino acid substitution most likely did not affect protein activity and stability (Table S5).

Single nucleotide polymorphisms unique for *H. heilmannii* isolate ASB1.4, not belonging to the wild-type population for spectinomycin, were also found in four different genes which are shown in Table S5. For 30S ribosomal protein S1 gene (*RpsA*), a SNP was present at codon 523 (GAG (glutamate) -> AAG (lysine)). Using BLAST and ConSurf, various amino acids were present at this position in other bacterial species, but never lysine. The Sorting Intolerant From Tolerant (SIFT) tool indicated that this amino acid substitution may affect the activity of *RpsA* with an accuracy of 46%, while the other tools predicted a neutral effect with an average accuracy of 76%. Using the I-Mutant 3.0 tool, the amino acid substitution did not seem to affect protein stability (DDG: −0.25, RI: 4). In addition, an SNP was found at codon 304 of the Ribosomal RNA small subunit methyltransferase D gene (*RsmD*) (ACC (threonine) -> GCG (alanine)). The BLAST analysis showed the presence of various amino acids at this position in other bacterial species and the ConSurf Server gave a conservation score below five. The MAPP tool indicated that this amino acid substitution may affect the activity of *RsmD* with an accuracy of 41%, while the other tools predicted a neutral effect with an average accuracy of 75%. The I-Mutant 3.0 tool showed that this amino acid substitution may decrease protein stability (DDG: −1.13, RI:9). Analysis of SNPs present in 30S ribosomal protein S12 (*RpsL*) and S7 (*RpsG*) genes indicated that these amino acid substitutions most likely did not affect protein activity and stability, Table S5).

Single nucleotide polymorphisms in ribosomal protein genes unique for *H. heilmannii* isolate ASB6.3, not belonging to the wild-type population for lincomycin, were not found.

### 3.4. Decreased Antimicrobial Susceptibility Mechanisms

Several SNPs were found in the 30S ribosomal protein genes of all *H. heilmannii* isolates showing high MIC values for neomycin, but not in *H. ailurogastricus* isolates showing low MIC values for this antimicrobial. The potential impact of these SNPs on protein activity and stability are shown in Table S5.

For example, an SNP was present at codon 1393 of the 30S ribosomal protein S1 gene (*RpsA*) (GAC (aspartate) -> GGC (glycine)). Using BLAST, various amino acids were present at the same position in other bacterial species, and the ConSurf Server gave a conservation score of five. The PredictSNP, PhD-SNP, PolyPhen-1, PolyPhen-2, and SNAP tools indicated that this amino acid substitution may affect the activity of *RpsA* with an average accuracy of 52%, while the other tools predicted a neutral effect with an average accuracy of 68%. Using the I-Mutant 3.0 tool, this substitution was predicted to decrease protein stability (DDG: −0.94, RI:2). Analysis of other SNPs present in the *RpsA* gene indicated that these amino acid substitutions could affect protein activity, although this was only observed for 1–3 PredictSNP tools, while the other tools indicated a neutral effect (Table S5).

*Helicobacter heilmannii* isolates also showed presence of a SNP in the 30S ribosomal protein S2 gene (*RpsB*) at codon 250, leading to an amino acid substitution from arginine (CGA) to glutamine (CAA). Using BLAST, various amino acids were present at these positions in other bacterial species and the ConSurf Server gave conservation score of 6. The PredictSNP, PolyPhen-1, PolyPhen-2, PhD-SNP, SIFT, SNAP, and PANTHER tools indicated that this amino acid substitution may affect the activity of RpsB with an average accuracy of 72%, while the MAPP tool predicted a neutral effect with an accuracy of 70%. Using the I-Mutant 3.0 tool, this substitution was predicted to decrease protein stability (DDG: −1.24, RI: 9). Other SNPs were also found, but most likely did not impact protein activity and stability of *RpsB*.

Several SNPs were present in the ribosomal RNA small subunit methyltransferase D (*RsmD*), for example at codon 217 (TTT (phenylalanine) -> GTG (valine)/ATT (isoleucine)), at codon 430 (CTA (leucine) -> GCA (alanine)) and at codon 527 (AAG (lysine) -> ACA (threonine)). Using BLAST, various amino acids were present at these position in other bacterial species, while the ConSurf Server gave conservation scores of at least seven. All the PredictSNP tools indicated that the amino acid substitution at codon 217 may affect the activity of *RsmD* with an average accuracy of 87%, while this was only indicated by 4 PredictSNP tools for the amino acid substitutions at codon 430 and 527. Using the I-Mutant 3.0 tool, the amino acid substitutions at codon 217, 430, and 527 were predicted to decrease the protein stability (DDG: −0.63, RI: 5; DDG: −1.48, RI: 8; DDG: −0.62, RI: 5, resp.). Analysis of other SNPs present in the *RsmD* gene indicated that these amino acid substitutions most likely did not affect protein activity and stability (Table S5).

*Helicobacter heilmannii* isolates also showed presence of a SNP in the ribosomal RNA small subunit methyltransferase H (*RsmH*) at codon 265, resulting in an amino acid substitution from isoleucine (ATT) to proline (CCA). Using BLAST, various amino acids were present at this position in other bacterial species and the ConSurf Server gave a conservation score of six. The PredictSNP, MAPP, PhD-SNP, SIFT, and PANTHER tools indicated that this amino acid substitution may affect the activity of *RsmH* with an average accuracy of 51%, while the other tools predicted a neutral effect with an average accuracy of 66%. Using the I-Mutant 3.0 tool, this amino acid substitution was predicted to decrease protein stability (DDG: −1.60, RI:6). Analysis of other SNPs present in the *RsmH* gene indicated that these amino acid substitutions could affect protein activity, although this was only observed for 1–3 PredictSNP tools, while the other tools indicated a neutral effect (Table S5).

An SNP was also present in the Ribosomal silencing factor (*RsfS*) gene at codon 25 (CTA (leucine) -> ATG (methionine)). Using BLAST, various amino acids were present at this position in other bacterial species and the ConSurf Server gave a conservation score below five. The PredictSNP, MAPP, PolyPhen-1, PolyPhen-2, and SIFT tools indicated that this amino acid substitution may affect *RsfS* activity, with an average accuracy of 52%, while the other tools indicated a neutral effect with an average accuracy of 75%. Using the I-Mutant 3.0 tool, this amino acid substitution was predicted to decrease protein stability (DDG: −1.01, RI:9). Analysis of other SNPs present in the *RsfS* gene indicated that these amino acid substitutions most likely did not affect protein activity and stability (Table S5).

Finally, several SNPs (*n* = 85) were found in the 30S ribosomal protein S15 (*RpsO*), S16 (*RpsP*), S21 (*RpsU*), S6 (*RpsF*), S9 (*RpsI*), ribosomal protein S12 methylthiotransferase (*RimO*), ribosomal RNA large subunit methyltransferase H (*RlmH* group_1616) and ribosomal RNA small subunit methyltransferase I (*RsmI*) genes of all *H. heilmannii* isolates showing high MIC values for neomycin, but analysis indicated that these amino acid substitutions most likely did not impact protein activity and stability (Table S5). No SNPs were detected in the 30S ribosomal protein genes S10 (*RpsJ*), S11 (*RpsK*), S12 (*RpsL*), S13 (*RpsM*), S14 type Z (*RpsZ*), S17 (*RpsQ*), S18 (*RpsR*), S19 (*RpsS*), S20 (*RpsT*), S3 (*RpsC*), S4 (*RpsD*), S5 (*RpsE*), S7 (*RpsG*), and S8 (*RpsH*), nor in ribosomal RNA small subunit methyltransferase genes E (*RsmE*), G (*RsmG*), and A (*RsmA* group 2011) of the *H. heilmannii* isolates (Table S6).

No unique SNPs in ribosomal protein genes were found for the all *H. ailurogastricus* strains (ASB7.1, ASB9.4, ASB11.2, ASB13.1, ASB21 and ASB23) showing higher wild-type MIC values for lincomycin compared to the *H. heilmannii isolates* (ASB1.4, ASB2.1, ASB3.2, ASB14.1, ASB19, and ASB20). Therefore, it cannot be concluded that there is a clear distinction between *H. heilmannii* and *H. ailurogastricus*, in contrast with neomycin where we can find a clear distinct distribution.

## 4. Discussion

Antimicrobial susceptibility testing of *H. pylori* was performed using the agar dilution method according to the CLSI guidelines [41]. However, since *H. heilmannii* and *H. ailurogastricus* can only be cultivated in a biphasic medium with acidic pH, we used an alternative method which has been shown feasible to determine the antimicrobial susceptibility of *H. suis* [21,27]. The results from this study demonstrate that the combined agar and broth dilution method followed by qPCR can be used for antimicrobial susceptibility testing of *H. heilmannii* and *H. ailurogastricus* as well. Nevertheless, this technique is most likely not feasible for clinical applications since the cultivation of *Helicobacter* spp. is laborious.

Compared to the CLSI standards, the MIC endpoints for both reference strains *E. coli* and *S. aureus* were significantly higher for aminoglycosides, fluoroquinolones, lincosamides, and macrolides when using the combined agar and broth dilution method at pH 5. For ceftiofur and ampicillin, antimicrobial activity was increased with acidic pH, as already described by CLSI [41]. These findings are in line with those of by Berlamont et al. [28], further indicating that medium composition and pH may influence antimicrobial activity in vitro [42]. Interpretation of MIC values of antimicrobial agents obtained in vitro may thus not always reflect their activity in vivo. In addition, the complexity of the gastric environment further complicates prediction of activity of these antimicrobials.

As described by Berlamont et al. [28], we mainly used the epidemiological criterion for interpretation of MICs to determine which isolates showed potential presence of acquired resistance [28,37]. Microorganisms without (i.e., wild type) and with acquired resistance mechanisms (i.e., non-wild type) to the antimicrobial agent in question are defined by this criterion, regardless of the clinical context (European Committee on Antimicrobial Susceptibility Testing (EUCAST), 2019). The presence of monomodal MIC distributions indicated that all *H. ailurogastricus* and the majority of *H. heilmannii* isolates fell within the wild-type range, whereas the presence of bimodal MIC distributions indicated that some *H. heilmannii* isolates (*n* = 5) that fell in the higher MIC range did not belong to the wild-type population. Using this epidemiological criterion does not guarantee the outcome of treatment of an infection with non-wild-type isolates and in vitro activity can still differ from in vivo activity. Still, by using this criterion, this may highlight that certain isolates have acquired mechanisms rendering them potentially less susceptible than the normal bacterial population to the antimicrobial agent tested. In this study, feline *H. heilmannii* isolates not belonging to the wild-type population were detected for azithromycin (one isolate), enrofloxacin (two isolates), lincomycin (one isolate), and spectinomycin (one isolate). Furthermore, *H. heilmannii* isolates showed higher MIC values for neomycin compared to *H. ailurogastricus* isolates. This indicates that acquired resistance and/or decreased susceptibility occasionally occur in *H. heilmannii* isolates. As pets may constitute a source of infection for humans, this should be kept in mind when dealing with a human patient infected with *H. heilmannii*.

Identifying presence of antimicrobial resistance mechanisms in *H. heilmannii* and *H. ailurogastricus* isolates not belonging to the wild type population is difficult due to the lack of available antimicrobial gene resistance databases for *Helicobacter* spp. As such, gene sequence comparison between isolates showing higher MIC values and the wild-type population was performed manually to detect potential presence of single nucleotide polymorphisms (SNPs). Several software tools were used to predict the impact of these SNPs on protein activity and stability (i.e., Jalview 2.10.5, ConSurf Server, I-Mutant 3.0 and PredictSNP) [39,40]. Further investigation remains warranted however, as the results from this study do not necessarily imply a causal relationship between the presence of antimicrobial resistance and the presence of SNPs.

*Helicobacter heilmannii* isolate ASB19 did not belong to the wild type population of azithromycin, indicating acquired resistance to this antimicrobial agent. This may be associated with the presence of SNPs in the *RplB* and *RplC* genes belonging to the 50S ribosomal protein family, as indicated by the different software tools for protein stability and activity (Table S5). Similarly, point mutations in these ribosomal protein encoding genes have also been associated with acquired resistance to macrolides in *Streptococcus criceti*, *S. pneumoniae*, *E. coli,* and *Brachyspira* spp. [43,44,45,46]. Cross-resistance to more than one macrolide has been described in several bacterial species, such as *Mycobacterium* sp. [47,48], but in this study, isolate ASB19 did not show cross-resistance to clarithromycin and tylosin.

Previous studies have shown presence of acquired resistance in two porcine *H. suis* isolates to fluoroquinolones (i.e., enrofloxcacin, levofloxacin, and moxifloxacin) [27,28]. Similarly, in this study, two feline *H. heilmannii* isolates (i.e., ASB14.1 and ASB20) did not belong to the wild-type population for enrofloxacin, indicating acquired resistance. In contrast with *H. suis*, however, this was not correlated with co-resistance to levofloxacin. For both *H. pylori* and *H. suis*, acquired resistance to fluoroquinolones has been associated with the presence of a point mutation at position 78 in the QRDR region of *gyrA* [27,49]. Conversely, we could not find presence of SNPs in the *gyrA* or *gyrB* genes of the *H. heilmannii* isolates. The mechanism involved in the acquired resistance of the two *H. heilmannii* isolates to enrofloxacin, therefore, remains unclear.

*Helicobacter heilmannii* isolate ASB1.4 did not belong to the wild-type population for spectinomycin, indicating presence of acquired resistance to this antimicrobial agent. Spectinomycin is an aminocyclitol, which, like aminoglycosides, inhibits protein synthesis via a 30S ribosomal target. In addition, ASB1.4 also showed higher MIC values for gentamicin (MIC value 32µg/mL) and neomycin (MIC value 8 µg/mL) compared to other *Helicobacter* spp. isolates, indicating decreased susceptibility to aminoglycosides (Table 1). Unique SNPs were found in the *RpsA*, *RpsL*, *RpsG,* and *RsmD* genes belonging to the 30S ribosomal protein gene family of *H. heilmannii* isolate ASB1.4. These SNPs may impact protein activity and stability, indicated by the different tools, and may be associated with the decreased susceptibility to the aminoglycosides and to spectinomycin. Similarly, point mutations in the *RpsA* gene have been associated with acquired resistance to aminoglycosides in *H. suis* [28], *M. tuberculosis* [50,51,52,53], and *E. coli* [54].

Compared to *H. ailurogastricus* isolates, *H. heilmannii* isolates showed higher wild-type MIC values for neomycin. Single nucleotide polymorphisms unique for *H. heilmannii* isolates were found in several 30S and 50S ribosomal protein encoding genes as well as in ribosomal RNA methyltransferase genes and might be associated with this decreased susceptibility, as indicated by the software tools. A different pattern of MIC distribution between the two *Helicobacter* species was also observed for lincomycin. For this antimicrobial agent, the *H. ailurogastricus* isolates showed higher wild type MIC values compared to the *H. heilmannii* isolates. No SNPs in ribosomal protein genes unique for *H. ailurogastricus* isolates were however found. In any case, due to the limited number of isolates tested, further studies are necessary to confirm differences in intrinsic susceptibility between *H. heilmannii* and *H. ailurogastricus* for neomycin and lincomycin.

The wild-type MIC values of ampicillin, clarithromycin, enrofloxacin, spectinomycin, neomycin, and oxytetracycline for *H. heilmannii* and *H. ailurogastricus* isolates were similar to those described for *H. pylori*, *H. bizzozeronii*, *H. felis* and *H. salomonis* [9,55], while higher wild-type MIC values were obtained for tylosin, lincomycin, and gentamicin and lower wild-type MIC values for metronidazole (Table S7). Nevertheless, antimicrobial susceptibility patterns of different gastric *Helicobacters* spp. are difficult to compare, as medium composition and pH variations can affect antimicrobial activity in vitro [41]. The only relevant species for comparison is *H. suis*, as we used the same enriched biphasic medium with acidic pH for *H. heilmannii* and *H. ailurogastricus*. Compared to *H. suis*, *H. heilmannii,* and *H. ailurogastricus* showed lower wild-type MIC values for β-lactams, gentamicin, rifamycins, tetracyclines, neomycin, and nitroimidazole (Table S7) [27,28] with similar wild-type MIC values for macrolides [28]. In general, *H. heilmannii* and *H. ailurogastricus* are more susceptible to antimicrobial agents compared to *H. suis* which may affect in vivo treatment.

In conclusion, acquired resistance to azithromycin, enrofloxacin, lincomycin, and spectinomycin seems to occasionally occur in feline *H. heilmannii* isolates. As pets may constitute a source of infection for humans, this should be taken into consideration when dealing with a human patient infected with *H. heilmannii*.

## Figures and Tables

**Table 1:** Detailed overview of MIC distribution of seven *H. heilmannii* and six *H. ailurogastricus* isolates. Feline *H. heilmannii* isolates: ASB1.4, ASB2.1, ASB3.2, ASB6.3, ASB14.1, ASB19, and ASB20. Feline *H. ailurogastricus* isolates: ASB7.1, ASB9.4, ASB11.2, ASB13.1, ASB21, and ASB23. *H. heilmannii* isolates showing acquired antimicrobial resistance are indicated in bold and red.

		Isolates with a MIC (μg/mL) of													
Group	Antimicrobial Agent	≤0.03125	0.0625	0.125	0.25	0.5	1	2	4	8	16	32	64	128	>128
**β-lactams**	**Ampicillin**			ASB3.2 ASB13.1	ASB1.4 ASB6.3 ASB23	ASB2.1 ASB14.1 ASB20 ASB11.2	ASB19 ASB7.1 ASB9.4 ASB21								
	**Ceftiofur**					ASB3.2	ASB20	ASB2.1 ASB19	ASB1.4 ASB6.3 ASB7.1 ASB9.4 ASB11.2 ASB13.1	ASB14.1 ASB23	ASB21				
**Macrolides**	**Clarithromycin**	ASB2.1	ASB3.2 ASB6.3 ASB21	ASB1.4 ASB14.1 ASB19 ASB7.1 ASB11.2 ASB13.1 ASB23	ASB20 ASB9.4										
	**Tylosin**					ASB20	ASB19 ASB11.2	ASB3.2 ASB21	ASB2.1 ASB6.3 ASB14.1 ASB7.1 ASB9.4 ASB13.1 ASB23	ASB1.4					
	**Azithromycin**	ASB2.1 ASB3.2 ASB6.3 ASB7.1 ASB9.4 ASB11.2	ASB20 ASB13.1 ASB23	ASB1.4 ASB21	ASB14.1				ASB19						
**Lincosamides**	**Lincomycin**					ASB1.4	ASB2.1 ASB3.2 ASB19	ASB14.1 ASB20	ASB7.1 ASB23	ASB13.1 ASB21	ASB9.4 ASB11.2	ASB6.3			
**Quinolones**	**Enrofloxacin**	ASB2.1 ASB23	ASB13.1	ASB1.4 ASB3.2 ASB19 ASB7.1 ASB11.2	ASB6.3 ASB9.4 ASB21				ASB14.1			ASB20			
	**Levofloxacin**	ASB1.4 ASB2.1 ASB3.2 ASB20 ASB7.1 ASB13.1 ASB21 ASB23	ASB19 ASB9.4 ASB11.2	ASB14.1	ASB6.3										
**Rifamycins**	**Rifampicin**	ASB2.1 ASB3.2 ASB6.3 ASB7.1 ASB11.2 ASB13.1	ASB1.4 ASB19 ASB20 ASB9.4 ASB21 ASB23	ASB14.1											
**Aminoglycosides**	**Gentamicin**							ASB2.1 ASB3.2 ASB14.1	ASB19 ASB7.1 ASB13.1 ASB21	ASB6.3 ASB20 ASB11.2 ASB23	ASB9.4	ASB1.4			
	**Neomycin**					ASB7.1 ASB9.4	ASB11.2	ASB2.1 ASB13.1 ASB21 ASB23	ASB14.1	ASB1.4	ASB3.2 ASB6.3 ASB20	ASB19			
**Aminocyclitol**	**Spectinomycin**	ASB2.1 ASB3.2	ASB7.1 ASB11.2 ASB13.1 ASB23	ASB6.3 ASB9.4	ASB20 ASB21	ASB19	ASB14.1				ASB1.4				
**Tetracyclines**	**Oxytetracycline**	ASB3.2 ASB20 ASB7.1 ASB9.4 ASB13.1 ASB23	ASB1.4 ASB11.2	ASB2.1 ASB19 ASB21	ASB6.3 ASB14.1										
	**Doxycycline**		ASB19 ASB21	ASB2.1 ASB3.2 ASB20 ASB7.1	ASB1.4 ASB6.3 ASB14.1 ASB9.4 ASB11.2 ASB13.1 ASB23										
**Nitroimidazole**	**Metronidazole**				ASB3.2 ASB23	ASB1.4 ASB2.1 ASB20 ASB7.1 ASB9.4 ASB13.1	ASB11.2 ASB21	ASB14.1	ASB6.3 ASB19						

## Supplementary Materials

The following are available online at https://www.mdpi.com/2076-2607/8/6/957/s1. Table S1: Antimicrobials agents used on the minimal inhibitory concentration determination in *H. heilmannii* and *H. ailurogastricus* species. Table S2: Primers used for the qPCR standards for *H. heilmannii* and *H. ailurogastricus*. Table S3: Detailed information of *Helicobacter* species used in the study. Table S4: Minimal inhibitory concentrations of *H. heilmannii* and *H. ailurogastricus* isolates (MIC range, average and standard deviation, and statistical analysis). Table S5: Feline *H. heilmannii* isolates included in analysis: ASB1.4, ASB2.1, ASB3.2, ASB6.3, ASB14.1, ASB19 and ASB20. Feline *H. ailurogastricus* isolates included in analysis: ASB7.1, ASB9.4, ASB11.2, ASB13.1, ASB21 and ASB23; Amino acid nomenclature: A: alanine, C: cysteine, D: aspartate, E: glutamate, F; phenylalanine, G: glycine, I: isoleucine, K: lysine, L: leucine, M: methionine, N: asparagine, P: proline, Q: glutamine, R: arginine, S: serine, T: threonine, V: valine; DDG: predicted free energy change; RI: relative index; CS: conservation score. Table S6: Genes investigated for the antimicrobial resistance mechanism with no SNPs founded. Table S7: Comparison of MIC values of *H. heilmannii* and *H. ailurogastricus* with others gastric *Helicobacter* sp.
