# Supplementary material for: Antimicrobial Susceptibility Pattern of Helicobacter heilmannii and Helicobacter ailurogastricus Isolates
Source: Microorganisms. 2020 Jun 25;8(6):957. doi: 10.3390/microorganisms8060957 (PMC7355750; doi:10.3390/microorganisms8060957)
Supplement: Supplementary file 1 [file microorganisms-08-00957-s001.pdf]

Supplementary Table S1. Antimicrobials agents used on the Minimal Inhibitory Concentration determination in *H. heilmannii* and *H. ailurogastricus* species.

Antimicrobial Agent	Code Sigma-Aldrich	Potency (µg/ml)
Ampicillin sodium salt	A9518	931,64
Azithromycin	75199	952,00
Ceftiofur hydrochloride (Pfizer)		
Clarithromycin	C9742	984,00
Doxycycline hyclate	D9891	987,04
Enrofloxacin	17849	998,00
Gentamycin sulfate	G1914	655,00
Levofloxacin	28266	999,00
Lincomycin hydrochloride	62143	919,68
Metronidazole	M1547	1000,00
Neomycin trisulfate salt hydrate	N1876	655,00
Oxytetracycline hydrochloride	O5875	950,37
Rifampicin	R3501	970,00
Spectinomycine dihydrochloride pentahydrate	S9007	667,00
Tylosin tartrate	T6271	936,00

Supplementary Table S2. Primers used for the qPCR standards for *H. heilmannii* and *H. ailurogastricus.*

**Specie**	**Primer Sequence**	
H. heilmannii	solU430F	5′- GCK GAW TTG ATG CAA GAA GG -3′
	sol1735R	5′- CTT CGT GRA TTT TAA RCC CAA T -3′
H. ailurogastricus	LpsA-ASB7-Fw	5′- CGA TCA AAG ATC GGG TGA AT -3′
	LpsA-ASB7-Rv	5′- CCA TTA AGG GGT GCT TGA AA -3′

Supplementary Table S3. Detailed information of *Helicobacter* species used in the study.

***Helicobacter* specie**	**Strain name**	**Strain ID**	**Host origin**	**Accesion Number**
H. heilmannii	ASB1.4	35817_16	Cat	CDMK00000000
H. heilmannii	ASB2.1	35817_7	Cat	CDMP00000000
H. heilmannii	ASB3.2	35817_8	Cat	CDMJ00000000
H. heilmannii	ASB6.3	35817_5	Cat	CDMM00000000
H. heilmannii	ASB14.1	35817_6	Cat	CDMI00000000
H. heilmannii	ASB19	35817_11	Cat	FZMG00000000
H. heilmannii	ASB20	35817_15	Cat	FZME00000000
H. ailurogastricus	ASB7.1	1578720_9	Cat	CDMG00000000
H. ailurogastricus	ASB9.4	1578720_10	Cat	CDMN00000000
H. ailurogastricus	ASB11.2	1578720_11	Cat	CDML00000000
H. ailurogastricus	ASB13.1	1578720_12	Cat	CDMH00000000
H. ailurogastricus	ASB21	1578720_3	Cat	FZLU00000000
H. ailurogastricus	ASB23	1578720_6	Cat	FZMH0000000

Supplementary Table S4. Minimal Inhibitory Concentrations of *H. heilmannii* and *H. ailurogastricus* isolates (MIC range, average and standard deviation, and statistical analysis). *H. heilmannii* isolates not belonging to the wild type population (ASB19 for azithromycin, ASB6.3 for lincomycin, ASB1.4 for spectinomycin and ASB14.1 and ASB20 for enrofloxacin) were not included on the average and standard deviation calculation, nor in the statistical analysis.

Group	Antimicrobial Agent	Specie	MIC range (µg/ml)	Average MIC value (µg/ml)	Standard Deviation (µg/ml)	Statistical Analysis (2-way ANOVA)
**β-lactams**	**Ampicillin**	*H. heilmannii*	0.125 - 1	0.45	0.29	ns (p>0.9999)
		H. ailurogastricus	0.125 - 1	0.65	0.41	
	**Ceftiofur**	*H. heilmannii*	0.5 - 8	3.07	2.56	ns (p=0.9993)
		H. ailurogastricus	4 - 16	6.67	4.84	
**Macrolides**	**Clarithromycin**	*H. heilmannii*	0.03125 – 0.25	0.11	0.07	ns (p>0.9999)
		H. ailurogastricus	0.0625 – 0.25	0.14	0.06	
	**Tylosin**	*H. heilmannii*	0.5 - 8	3.36	2.53	ns (p>0.9999)
		H. ailurogastricus	1 - 4	3.17	1.33	
	**Azithromycin**	*H. heilmannii*	0.03125 – 0.25 (exception of ASB19– MIC=4)	0.09	0.09	ns (p>0.9999))
		H. ailurogastricus	0.03125 – 0.125	0.06	0.04	
**Lincosamides**	**Lincomycin**	*H. heilmannii*	0.5 – 2 (exception of ASB6.3 – MIC=32)	1.25	0.61	ns (p=0.6301)
		H. ailurogastricus	4 - 16	9.33	5.47	
**Quinolones**	**Enrofloxacin**	*H. heilmannii*	0.03125 – 0.25 (exception of ASB14.1 and ASB20 – MIC=4 and 32)	0.13	0.08	ns (p>0.9999)
		H. ailurogastricus	0.03125 – 0.25	0.14	0.09	
	**Levofloxacin**	*H. heilmannii*	0.03125 – 0.25	0.08	0.08	ns (p>0.9999)
		H. ailurogastricus	0.03125 – 0.0625	0.04	0.02	
**Rifamycins**	**Rifampicin**	*H. heilmannii*	0.03125 – 0.125	0.06	0.03	ns (p>0.9999)
		H. ailurogastricus	0.03125 – 0.0625	0.05	0.02	
**Aminoglycosides**	**Gentamicin**	*H. heilmannii*	2 - 32	8.29	10.8	ns (p>0.9999)
		H. ailurogastricus	4 - 16	7.33	4.68	
	**Neomycin**	*H. heilmannii*	2 - 32	13.43	10.11	ns (p=0.1317)
		H. ailurogastricus	0.5 - 2	1.33	0.75	
**Aminocyclitol**	**Spectinomycin**	*H. heilmannii*	0.03125 – 1	0.32	0.38	ns

			(exception of ASB1.4 – MIC=16)			(p>0.9999)
		H. ailurogastricus	0.0625 – 0.25	0.10	0.08	
**Tetracyclines**	**Oxytetracycline**	*H. heilmannii*	0.03125 – 0.25	0.13	0.09	ns
		H. ailurogastricus	0.03125 – 0.125	0.05	0.04	(p>0.9999)
	**Doxycycline**	*H. heilmannii*	0.0625 – 0.25	0.17	0.08	ns
		H. ailurogastricus	0.0625 – 0.25	0.20	0.08	(p>0.9999)
**Nitroimidazole**	**Metronidazole**	*H. heilmannii*	0.25 - 4	1.68	1.69	ns
		H. ailurogastricus	0.25 – 0.5	0.63	0.31	(p>0.9999)

Supplementary Table S5. Feline *H. heilmannii* isolates included in analysis: ASB1.4, ASB2.1, ASB3.2, ASB6.3, ASB14.1, ASB19 and ASB20. Feline *H. ailurogastricus* isolates included in analysis: ASB7.1, ASB9.4, ASB11.2, ASB13.1, ASB21 and ASB23; Amino acid nomenclature: A: alanine, C: cysteine, D: aspartate, E: glutamate, F; phenylalanine, G: glycine, I: isoleucine, K: lysine, L: leucine, M: methionine, N: asparagine, P: proline, Q: glutamine, R: arginine, S: serine, T: threonine, V: valine; DDG: predicted free energy change; RI: relative index; CS: conservation score.

**SNPs related to**	**Location**		**Codon and corresponding amino acid**		**Impact SNPs on protein activity/stability**			
	**Gene**	**Codon**	**non-wild type isolate(s)**	**wild type isolate(s)**	**amino acid characteristics**	**predictSNP (accuracy %)**	**I-Mutant 3.0**	**ConSurf**
**acquired azithromycin resistance (macrolide) in ASB19 (*H. heilmannii*)**	*50S ribosomal protein L2 (RplB)*	613	AGC -> S	AAC -> N	S: polar<br>N: neutral	all tools:<br>neutral (83%)	DDG: -0.44,<br>RI: 3	other species:<br>various amino acids<br>CS: 1
	50S ribosomal protein L3 (RplC)	259	TCT -> S	CCT -> P	S: polar<br>P: aromatic side chain	all tools:<br>neutral (83%)	DDG: -1.31,<br>RI: 8	other species:<br>various amino acids<br>CS: 1
		277	TGC -> C	CGC -> R	C: polar, S-group<br>R: + charge	MAPP tool:<br>deleterious (41%)<br>PhD-SNP tool:<br>deleterious (68%)<br>PolyPhen-1:<br>deleterious (59%)<br>other tools:<br>neutral (60%)	DDG: -0.64,<br>RI: 0	other species:<br>various amino acids, but never C<br>CS: 1
**acquired spectinomycin (aminoglycoside) resistance in ABS1.4**	*30S ribosomal protein S1 (RpsA)*	523	AAG -> K	GAG -> E	K: + charge<br>E: - charge	SIFT tool:<br>deleterious (46%)<br>other tools:<br>neutral (74%)	DDG: -0.25,<br>RI: 4	other species: E, A, G, Q, S, T, L, D, N, R, but never K<br>CS: 1
	30S ribosomal protein S12 (RpsL)	391	GAC -> D	GAA -> E	D: - charge<br>E: - charge	all tools:<br>neutral (83%)	DDG: 0.02,<br>RI: 1	other species: E, K, G, S, A, but never D<br>CS: 1
	30S ribosomal protein S7 (RpsG)	64	GTA -> V	ATC -> I	V: no S-group<br>I: neutral	all tools:<br>neutral (83%)	DDG: -1.05,<br>RI: 7	other species: V, L, I, T, A, F<br>CS: 7

	Ribosomal RNA small subunit methyltransferase D (RsmD)	304	GCG -> A	ACC -> T	A: neutral, hydrophobic side chain, non-polar<br>T: polar	MAPP tool: deleterious (41%)<br>all tools: neutral (75%)	DDG: -1.13, RI: 9	other species: T, M, S, I, F, P, A, L, C, V<br>CS: 4
**higher MICs for neomycin in all *H. heilmannii* strain isolates**	*30S ribosomal protein S1 (RpsA)*	31	CAT-> H	CAA-> Q	H: + charge<br>Q: neutral, non-polar	PolyPhen-1: deleterious (74%)<br>PolyPhen-2: deleterious (54%)<br>other tools: neutral (63%)	DDG: -0.54, RI: 2	other species: various amino acids<br>CS: 1
		73	GAG -> E	GGG-> G	E: - charge<br>G: neutral	all tools: neutral (83%)	DDG: -0.07, RI: 3	other species: various amino acids<br>CS: 5
		118	GAG-> D	GGG-> E	D: - charge<br>E: - charge	all tools: neutral (83%)	DDG: -0.23, RI: 5	other species: various amino acids<br>CS: 1
		226	CAG-> E	GAC-> Q	E: - charge<br>Q: neutral	all tools: neutral (83%)	DDG: -0.38, RI: 4	other species: various amino acids<br>CS: 1
		238	GGA-> G	GCA-> A	G: neutral<br>A: neutral, hydrophobic side chain, non-polar	all tools: neutral (83%)	DDG: -1.33, RI: 9	other species: various amino acids<br>CS: 2
		241	CTC-> V	GTA-> L	L/ V: neutral, hydrophobic side chain	PANTHER tool: deleterious (57%)<br>other tools: neutral (83%)	DDG: -1.79, RI: 9	other species: various amino acids<br>CS: 1
		250	CCC-> T	ACG-> P	T: polar<br>P: aromatic side chain	PANTHER tool: deleterious (61%)<br>other tools: neutral (83%)	DDG: -1.43, RI: 9	other species: various amino acids<br>CS: 1
		271	TTA-> Q	CAA-> L	Q: neutral	PhD-SNP tool: deleterious (59%)	DDG: -2.31, RI: 9	other species: various amino acids<br>CS: 1

					L: neutral, hydrophobic side chain	PANTHER tool: deleterious (65%) other tools: neutral (74%)		
		274	AGA-> K	AAG-> R	K/R: + charge	all tools: neutral (83%)	DDG: -1.04, RI: 9	other species: various amino acids CS: 4
		343	ACT-> S	TCT-> T	S/T: polar	all tools: neutral (83%)	DDG: -0.62, RI: 6	other species: various amino acids CS: 1
		352	GCG-> G	GGA-> A	G: neutral A: neutral, hydrophobic side chain, non-polar	SIFT tool: deleterious (53%) other tools: neutral (74%)	DDG: -1.21, RI: 8	other species: various amino acids CS: 1
		364	GAC-> E	GAA-> D	E/D: - charge	all tools: neutral (83%)	DDG: -1.02, RI: 5	other species: various amino acids CS: 2
		370	TTT-> I	ATC-> F	I: neutral F: non-polar	PANTHER tool: deleterious (65%) other tools: neutral (83%)	DDG: -0.90, RI: 9	other species: various amino acids CS: 1
		385	ATC-> V	GTC-> I	V: neutral, hydrophobic side chain I: neutral	all tools: neutral (83%)	DDG: -0.90, RI: 4	other species: V, I CS: 6
		388	GTT-> A	GTC-> V	A: neutral, hydrophobic side chain, non-polar V: neutral, hydrophobic side chain	SIFT tool: deleterious (53%) other tools: neutral (74%)	DDG: -1.54, RI: 9	other species: various amino acids CS: 3
		412	GTT-> I	ATC-> V	I: neutral	all tools: neutral (83%)	DDG: -0.36, RI: 6	other species: various amino acids CS: 4

					V: neutral, hydrophobic side chain			
		418	ATC-> V	GTG-> I	V: neutral, hydrophobic side chain<br>I: neutral	all tools: neutral (83%)	DDG: -0.94, RI: 6	other species: various amino acids<br>CS: 1
		424	CAT-> N	AAT-> H	N: neutral, polar<br>H: + charge	PhD-SNP tool: deleterious (58%)<br>Other tools: neutral (74%)	DDG: -0.74, RI: 4	other species: various amino acids<br>CS: 1
		427	CAG-> E	GAG-> Q	E: - charge<br>Q: neutral	all tools: neutral (83%)	DDG: -0.18, RI: 2	other species: various amino acids<br>CS: 2
		532	AGC-> N	AAC-> S	N: neutral, polar<br>S: polar	all tools: neutral (83%)	DDG: -0.67, RI: 1	other species: various amino acids<br>CS: 2
		543	TCT-> A	GCG-> S	A: neutral, hydrophobic side chain, non-polar<br>S: polar	MAPP tool: deleterious (62%)<br>PhD-SNP tool: deleterious (58%)<br>Other tools: neutral (63%)	DDG: -0.94, RI: 8	other species: various amino acids<br>CS: 5
		547	GTT-> I	ATC-> V	I: neutral<br>V: neutral, hydrophobic side chain, non-polar	Poly-Phen-2 tool: deleterious (47%)<br>PANTHER tool: deleterious (61%)<br>Other tools: neutral (74%)	DDG: -0.56, RI: 4	other species: various amino acids<br>CS: 5
		568	GAG-> D	GAT-> E	D/E: - charge	PhD-SNP tool: deleterious (86%)<br>Poly-Phen-2 tool: deleterious (43%)	DDG: -0.45, RI: 2	other species: various amino acids<br>CS: 5

						PANTHER tool: deleterious (57%) Other tools: neutral (63%)		
		571	GTC-> I	ATT-> V	I: neutral V: neutral, hydrophobic side chain, non-polar	PANTHER tool: deleterious (61%) Other tools: neutral (81%)	DDG: -0.26, RI: 0	other species: various amino acids CS: 2
		580	AAA-> K	CGA-> R	K/R: + charge	all tools: neutral (83%)	DDG: -0.79, RI: 8	other species: various amino acids CS: 1
		1111	GAA-> E	GAT-> D	E/D: - charge	all tools: neutral (83%)	DDG: -0.48, RI: 0	other species: various amino acids CS: 1
		1129	CGC-> R	CAC-> H	R/H: + charge	PANTHER tool: deleterious (61%) other tools: neutral (83%)	DDG: -0.05, RI: 2	other species: various amino acids CS: 5
		1171	AAC-> N	GAT-> D	N: neutral, polar D: - charge	PhD-SNP tool: deleterious (59%) Poly-Phen-2 tool: deleterious (50%) nsSNPAnalyzer tool: deleterious (63%) Other tools: neutral (65%)	DDG: -0.72, RI: 3	other species: various amino acids CS: 6
		1246	CAA-> Q	AAA-> K	Q: neutral K: + charge	Poly-Phen-2 tool: deleterious (47%) PANTHER tool: deleterious (57%) Other tools: neutral (74%)	DDG: -0.23, RI: 1	other species: various amino acids CS: 1

		1258	GAC-> D	GAA-> E	D/E: - charge	all tools: neutral (83%)	DDG: -0.29, RI: 4	other species: various amino acids CS: 3
		1285	AAA-> K	CAA-> Q	K: + charge<br>Q: neutral	all tools: neutral (83%)	DDG: -0.29, RI: 2	other species: various amino acids CS: 6
		1288	ACC-> T	GTC-> V	T: polar<br>V: neutral, hydrophobic side chain, non-polar	MAPP tool: deleterious (51%)<br>SIFT tool: deleterious (45%)<br>PANTHER tool: deleterious (71%)<br>Other tools: neutral (65%)	DDG: -0.91, RI: 9	other species: V, A. F, T<br>CS: 8
		1306	GAC-> D	AAC-> N	D: - charge<br>N: neutral, polar	PANTHER tool: deleterious (68%)<br>Other tools: neutral (83%)	DDG: 0.08, RI: 1	other species: various amino acids CS: 8
		1312	GAC-> D	GAA-> E	D/E: - charge	all tools: neutral (83%)	DDG: -0.03, RI: 2	other species: various amino acids CS: 1
		1315	AAT-> N	GAT-> D	N: neutral, polar<br>D: - charge	all tools: neutral (83%)	DDG: -0.78, RI: 5	other species: various amino acids CS: 3
		1342	TAC-> Y	TTT-> F	Y: polar<br>F: non-polar, no S-group	MAPP tool: deleterious (59%)<br>Other tools: neutral (74%)	DDG: -0.76, RI: 0	other species: various amino acids CS: 1
		1351	GCC-> A	CCT-> P	A: neutral, hydrophobic side chain, non-polar<br>P: aromatic side chain	all tools: neutral (83%)	DDG: -1.16, RI: 8	other species: various amino acids CS: 1

		1360	ACC-> T	GTC-> V	T: polar<br>V: neutral, hydrophobic side chain, non-polar	all tools: neutral (83%)	DDG: -1.03, RI: 9	other species: various amino acids<br>CS: 5
		1384	AAA-> K	CGC-> R	R/K: + charge	all tools: neutral (83%)	DDG: -0.53, RI: 7	other species: various amino acids<br>CS: 1
		1393	GGC-> G	GAC-> D	G: neutral<br>D: -charge	PredictSNP tool: deleterious (52%)<br>PhD-SNP tool: deleterious (59%)<br>PolyPhen-1 tool: deleterious (43%)<br>PolyPhen-2 tool: deleterious (53%)<br>SNAP tool: deleterious (56%)<br>Other tools: neutral (67%)	DDG: -0.94, RI: 2	other species: various amino acids<br>CS: 5
		1399	ATT-> I	GTT-> V	V/I: neutral, hydrophobic side chain	all tools: neutral (83%)	DDG: -0.29, RI: 5	other species: M, I, A, V, L<br>CS: 7
		1408	AAG-> K	ACA-> T	K: + charge<br>T: polar	PhD-SNP tool: deleterious (59%)<br>other tools: neutral (74%)	DDG: -0.66, RI: 5	other species: various amino acids<br>CS: 1
		1435	ATT-> I	GTC-> V	V/I: neutral, hydrophobic side chain	all tools: neutral (83%)	DDG: -0.68, RI: 8	other species: M, I, A, L, V<br>CS: 4
		1444	AGT-> S	GGC-> G	S: polar<br>G: neutral	PhD-SNP tool: deleterious (73%)	DDG: -1.10, RI: 6	other species: various amino acids<br>CS: 2

						other tools: neutral (75%)		
		1456	GTT-> V	ATT-> I	I/V: neutral, hydrophobic side chain	all tools: neutral (83%)	DDG: -0.73, RI: 4	other species: Y. L, V. M, I<br>CS: 6
		1474	GCC-> A	TCT-> S	A: neutral, hydrophobic side chain, non-polar<br>S: polar	MAPP tool: deleterious (41%)<br>other tools: neutral (74%)	DDG: -0.39, RI: 9	other species: various amino acids<br>CS: 4
		1483	ATC-> I	CTC-> L	L/I: neutral, hydrophobic side chain	all tools: neutral (83%)	DDG: -0.72, RI: 6	other species: M, I, Y, F, V, L<br>CS: 6
		1486	TAC-> Y	CAC-> H	Y: polar<br>H: + charge	all tools: neutral (83%)	DDG: 0.29, RI: 7	other species: various amino acids<br>CS: 1
		1522	ATG-> M	GTC-> V	M: non-polar<br>V: neutral, hydrophobic side chain	SIFT tool: deleterious (53%)<br>other tools: neutral (74%)	DDG: -0.83, RI: 7	other species: various amino acids<br>CS: 1
		1525	GTG-> V	ATT-> I	I/V: neutral, hydrophobic side chain	all tools: neutral (83%)	DDG: -0.69, RI: 4	other species: I, V, L, F<br>CS: 7
		1531	AGC-> S	GGT-> G	S: polar<br>G: neutral	PhD-SNP tool: deleterious (61%)<br>other tools: neutral (74%)	DDG: -1.19, RI: 9	other species: S. C, A, G<br>CS: 5
		1555	GGC-> G	GCC-> A	G: neutral<br>A: neutral, hydrophobic side chain, non-polar	SIFT tool: deleterious (46%)<br>other tools: neutral (74%)	DDG: -1.29, RI: 7	other species: various amino acids<br>CS: 1
		1594	CAC-> H	CGC-> R	R/H: + charge	all tools: neutral (83%)	DDG: -1.17, RI: 9	other species: various amino acids

								CS: 3
		1630	GCC-> A	TCG-> S	A: neutral, hydrophobic side chain, non-polar<br>S: polar	all tools: neutral (83%)	DDG: -0.22, RI: 5	other species: various amino acids<br>CS: 1
		1633	AAA-> K	AGC-> S	K: + charge<br>S: polar	PhD-SNP tool: deleterious (61%)<br>Other tools: neutral (74%)	DDG: -0.07, RI: 5	other species: various amino acids<br>CS: 1
		1639	AGC-> S	ACC-> T	T/S: polar	all tools: neutral (83%)	DDG: -0.61, RI: 4	other species: various amino acids<br>CS: 1
		1666	GGC-> G	AGC-> S	G: neutral<br>S: polar	all tools: neutral (83%)	DDG: -0.74, RI: 7	other species: various amino acids<br>CS: 3
	30S ribosomal protein S15 (RpsO)	73	AGC -> S	TGT -> C	S: polar<br>C: polar, S-group	PhD-SNP tool: deleterious (59%)<br>Other tools: neutral (74%)	DDG: -0.70, RI: 6	other species: various amino acids<br>CS: 3
		121	GCC -> A	GTC -> V	A/V: neutral, hydrophobic side chain, non-polar	MAPP tool: deleterious (51%)<br>Other tools: neutral (74%)	DDG: -1.43, RI: 7	other species: various amino acids<br>CS: 4
		133	AAC -> N	GAC -> D	N: neutral, polar<br>D: - charge	all tools: neutral (83%)	DDG: -0.88, RI: 6	other species: various amino acids<br>CS: 1
		214	GAC -> D	AAC-> N	D: - charge<br>N: neutral, polar	MAPP tool: deleterious (59%)<br>Other tools: neutral (74%)	DDG: -0.19, RI: 1	other species: various amino acids<br>CS: 1
		238	ACA -> T	GAG -> E	T: polar<br>E: - charge	all tools: neutral (83%)	DDG: 0.07, RI: 1	other species: various amino acids<br>CS: 1

	30S ribosomal protein S16 (RpsP)	127	GCA -> A	AGC -> S	A: hydrophobic side chain<br>S: polar	all tools: neutral (83%)	DDG: -0.90, RI: 8	other species: various amino acids<br>CS: 2
		130	AGC -> S	AAC -> N	S: polar<br>N: neutral, polar	MAPP tool: deleterious (62%)<br>PANTHER tool: deleterious (57%)<br>Other tools: neutral (73%)	DDG: -0.40, RI: 5	other species: various amino acids<br>CS: 1
		157	GCG -> A	GAG -> E	A: hydrophobic side chain<br>E: polar, - charge	MAPP tool: deleterious (57%)<br>PhD-SNP tool: deleterious (73%)<br>PANTHER tool: deleterious (68%)<br>Other tools: neutral (63%)	DDG: -0.40, RI: 5	other species: various amino acids<br>CS: 5
		166	GAC -> D	ACG -> S	D: - charge<br>S: polar	all tools: neutral (83%)	DDG: -0.14, RI: 2	other species: various amino acids<br>CS: 2
	30S ribosomal protein S2 (RpsB)	250	CAA -> Q	CGA -> R	Q: non-polar, neutral<br>R: + charge	Other tools: deleterious (72%)<br>MAPP tool: neutral (70%)	DDG: -1.24, RI: 9	other species: various amino acids<br>CS: 6
		694	ACG -> T	GCG -> A	T: polar<br>A: hydrophobic side chain, non-polar	all tools: neutral (83%)	DDG: -0.55, RI: 4	other species: various amino acids<br>CS: 1
		697	ATC -> I	GTT -> V	V/I: neutral, hydrophobic	all tools: neutral (83%)	DDG: -0.35, RI: 5	other species: various amino acids<br>CS: 1
		700	AAA -> K	GAG -> E	K: polar, + charge<br>E: polar, - charge	all tools: neutral (83%)	DDG: -0.66, RI: 8	other species: various amino acids<br>CS: 1

		709	GAG -> E	CAG-> Q	E: polar, - charge<br>Q: non-polar, neutral	all tools: neutral (83%)	DDG: -0.05, RI: 6	other species: various amino acids<br>CS: 5
		739	ATC -> I	GTC -> V	V/I: neutral, hydrophobic	all tools: neutral (83%)	DDG: -0.03, RI: 2	other species: various amino acids<br>CS: 1
		772	GTG -> V	GCA-> A	V/A: neutral, hydrophobic	MAPP tool: deleterious (46%)<br>other tools: neutral (83%)	DDG: 0.05, RI: 3	other species: K, V, A, T, E<br>CS: 6
		775	GAA -> E	GAC -> D	E: polar, - charge<br>D: - charge	all tools: neutral (83%)	DDG: 0.73, RI: 8	other species: E, D<br>CS: 7
	30S ribosomal protein S21 (RpsU)	202	TCT -> S	GCG -> A	S: polar<br>A: hydrophobic side chain, non-polar	all tools:<br>neutral (85%)	DDG: -0.71, RI: 8	other species: E, S, A<br>CS: 5
	30S ribosomal protein S6 (RpsF)	61	AGC -> S	AAC-> N	S: polar<br>N: neutral, polar	all tools:<br>neutral (83%)	DDG: 0.05, RI: 3	other species: various amino acids<br>CS: 4
		94	AAT-> N	GGA -> G	N: neutral, polar<br>G: non-polar, no S-group	all tools:<br>neutral (83%)	DDG: -0.87, RI: 6	other species: various amino acids<br>CS: 1
		175	TAC -> Y	CAC -> H	Y: polar<br>H: + charge	all tools:<br>neutral (83%)	DDG: 0.51, RI: 7	other species: various amino acids<br>CS: 5
		208	CAG -> Q<br>GCA -> A	AAA -> K	Q: neutral<br>A: hydrophobic side chain, non-polar<br>K: + charge	MAPP tool: deleterious (41%)<br>other tools: neutral (74%)	DDG: -0.34, RI: 4	other species: various amino acids<br>CS: 1
	30S ribosomal protein S9 (RpsI)	4	GCA -> A	ACA -> T	A: hydrophobic side chain, non-polar<br>T: polar	all tools:<br>neutral (83%)	DDG: -1.00, RI: 9	other species: T, A, V, S, E<br>CS: 6
		271	ACG -> T	AGT-> S	T/S: polar	PhD-SNP tool: deleterious (59%)	DDG: -0.01, RI: 5	other species: various amino acids<br>CS: 1

						SIFT tool: deleterious (46%) other tools: neutral (65%)		
	Ribosomal protein S12 methylthiotransferase (RimO)	808	TCC -> S	GCA -> A<br>ACC -> T	S: polar<br>A: hydrophobic side chain, non-polar<br>T: polar	PANTHER tool: deleterious (66%) other tools: neutral (83%)	DDG: -0.18, RI: 4	other species: various amino acids CS: 2
		901	AGC -> S	AGG -> R	S: polar<br>R: + charge	PANTHER tool: deleterious (66%) other tools: neutral (83%)	DDG: -0.69, RI: 6	other species: various amino acids CS: 4
		919	GAG -> E	CAA -> Q	E: polar, + charge<br>Q: neutral	MAPP tool: deleterious (43%) SIFT tool: deleterious (43%) PANTHER tool: deleterious (69%) other tools: neutral (64%)	DDG: -0.05, RI: 3	other species: various amino acids CS: 1
		922	GCG-> A	CAG -> E	A: hydrophobic side chain, non-polar<br>E: polar, + charge	MAPP tool: deleterious (48%) other tools: neutral (74%)	DDG: -0.29, RI: 6	other species: various amino acids CS: 1
		931	CAA -> Q	GAG -> E	Q: neutral<br>E: polar, + charge	PANTHER tool: deleterious (66%) other tools: neutral (83%)	DDG: -0.40, RI: 4	other species: various amino acids CS: 1
		934	GAG -> E	GGC -> G	E: polar, + charge<br>G: non-polar, no S-group	PANTHER tool: deleterious (76%) other tools: neutral (83%)	DDG: -0.33, RI: 1	other species: various amino acids CS: 4
		940	CAA -> Q<br>AGA -> R	ACA -> T	Q: neutral<br>R: + charge	PolyPhen-2 tool: deleterious (67%)	DDG: -0.52, RI: 4	other species: various amino acids

					T: polar	PANTHER tool: deleterious (74%)<br>other tools: neutral (74%)		CS: 3
		988	AAA -> K	AGG -> R	K/R: + charge	PANTHER tool: deleterious (61%)<br>other tools: neutral (83%)	DDG: -0.38, RI: 5	other species: various amino acids<br>CS: 1
		1015	GAC -> D<br>AGC -> S<br>AAC -> N	CCT -> P	D: - charge<br>S: polar<br>N: neutral, polar<br>P: aromatic side chain	PhD-SNP tool: deleterious (68%)<br>other tools: neutral (75%)	DDG: -1.35, RI: 8	other species: various amino acids<br>CS: 4
		1030	GAG -> E	GCC -> A	E: polar, + charge<br>A: hydrophobic side chain, non-polar	PANTHER tool: deleterious (69%)<br>other tools: neutral (83%)	DDG: -0.60, RI: 6	other species: various amino acids<br>CS: 1
		1036	CTT -> L	ATC -> I	L/I: neutral, hydrophobic side chain	PANTHER tool: deleterious (71%)<br>other tools: neutral (83%)	DDG: 0.03, RI: 1	other species: various amino acids<br>CS: 3
		1063	GAG-> E	AAA -> K	E: polar, + charge<br>K: + charge	PANTHER tool: deleterious (61%)<br>other tools: neutral (83%)	DDG: -0.76, RI: 7	other species: various amino acids<br>CS: 1
		1075	GCT -> A<br>ACT -> V	ATG -> M	A: hydrophobic side chain, non-polar<br>V: neutral, hydrophobic<br>M: non-polar	MAPP tool: deleterious (48%)<br>PANTHER tool: deleterious (74%)<br>other tools: neutral (83%)	DDG: -0.57, RI: 3	other species: various amino acids<br>CS: 2
		1087	AGT -> S<br>AAC -> N	GCT -> A	S: polar<br>N: neutral, polar	PANTHER tool: deleterious (74%)<br>other tools:	DDG: -0.34, RI: 7	other species: various amino acids<br>CS: 1

					A: hydrophobic side chain, non-polar	neutral (83%)		
		1210	ATG -> M<br>GTG -> V	TCT -> S	M: non-polar<br>V: neutral, hydrophobic<br>S: polar	all tools:<br>neutral (83%)	DDG: 0.20,<br>RI: 1	other species: various amino acids<br>CS: 1
		1246	CAC -> H	CTT -> L	H: + charge<br>L: neutral, hydrophobic side chain	all tools:<br>neutral (83%)	DDG: -1.50,<br>RI: 6	other species: various amino acids<br>CS: 1
		1252	CAC -> H	ACC -> T	H: + charge<br>T: polar	PhD-SNP tool:<br>deleterious (59%)<br>PolyPhen-1 tool:<br>deleterious (74%)<br>PolyPhen-2 tool:<br>deleterious (40%)<br>other tools:<br>neutral (60%)	DDG: -0.82,<br>RI: 5	other species: T<br>various amino acids<br>CS: 7
		1255	AGC -> S	ACT -> T	S/T: polar	all tools:<br>neutral (83%)	DDG: -0.21,<br>RI: 1	other species: various amino acids<br>CS: 1
		1258	TTT -> F	TGC -> C	F: non-polar, no S-group<br>C: polar, S-group	PhD-SNP tool:<br>deleterious (59%)<br>other tools:<br>neutral (74%)	DDG: 0.43,<br>RI: 2	other species: C, S, I, F, A<br>CS: 5
		1261	GAA -> E	CAA -> Q	A: hydrophobic side chain, non-polar<br>T: polar	all tools:<br>neutral (83%)	DDG: 0.07,<br>RI: 4	other species: various amino acids<br>CS: 1
		1270	ATA -> I	ATG-> M	I: neutral, hydrophobic side chain<br>M: non-polar	all tools:<br>neutral (83%)	DDG: -0.17,<br>RI: 5	other species: M, F, I, E, D, S<br>CS: 6

		1276	CTG -> L	GTG -> V	L: neutral, hydrophobic side chain<br>V: neutral, hydrophobic	all tools:<br>neutral (83%)	DDG: -0.56,<br>RI: 5	other species: Y, F, I, L, V<br>CS: 5
		1282	AGG -> R	AAG -> K	R/K: + charge	all tools:<br>neutral (83%)	DDG: 0.20,<br>RI: 1	other species: E, R, K<br>CS: 5
	Ribosomal RNA large subunit methyltransferase H (RlmH group_1616)	25	TCT -> S	GCC -> A	S: polar<br>A: hydrophobic side chain, non-polar	all tools:<br>neutral (83%)	DDG: -0.75,<br>RI: 8	other species: various amino acids<br>CS: 5
		34	ACC -> T	AAC -> N	T: polar<br>N: neutral, polar	MAPP tool:<br>deleterious (46%)<br>other tools:<br>neutral (74%)	DDG: 0.32,<br>RI: 2	other species: various amino acids<br>CS: 3
		40	GAG -> E	GAT -> D	E: polar, + charge<br>D: - charge	all tools:<br>neutral (83%)	DDG: 0.35,<br>RI: 4	other species: various amino acids<br>CS: 1
		88	AGA -> R	AAG -> K	R/K: + charge	all tools:<br>neutral (83%)	DDG: 0.22,<br>RI: 2	other species: K, G, R, N<br>CS: 5
		106	ATA -> I	GTC -> V	V/I: neutral, hydrophobic	all tools:<br>neutral (83%)	DDG: 0.03,<br>RI: 2	other species: various amino acids<br>CS: 1
		115	GCG -> A	TTT -> F	A: hydrophobic side chain, non-polar<br>F: non-polar, no S-group	MAPP tool:<br>deleterious (72%)<br>SIFT tool:<br>deleterious (79%)<br>other tools:<br>neutral (63%)	DDG: -2.28,<br>RI: 8	other species: various amino acids<br>CS: 6
		127	TCA -> S	TTG -> L	S: polar<br>L: neutral, hydrophobic side chain	PolyPhen-2 tool:<br>deleterious (40%)<br>SIFT tool:<br>deleterious (53%)	DDG: -2.29,<br>RI: 9	other species: L, I, S, V<br>CS: 8

						other tools:<br>neutral (68%)		
		130	AAC -> N	ACA -> T	N: neutral, polar<br>T: polar	MAPP tool:<br>deleterious (75%)<br>other tools:<br>neutral (75%)	DDG: -1.31,<br>RI: 7	other species:<br>various amino acids<br>CS: 2
		139	ACA -> T	TCC -> S	T/S: polar	MAPP tool:<br>deleterious (59%)<br>other tools:<br>neutral (74%)	DDG: -0.12,<br>RI: 5	other species:<br>various amino acids<br>CS: 1
		142	GAG -> E	AAT -> N	E: polar, + charge<br>N: neutral, polar	MAPP tool:<br>deleterious (51%)<br>other tools:<br>neutral (74%)	DDG: 0.25,<br>RI: 7	other species:<br>various amino acids<br>CS: 4
		148	CAA -> Q	AAG -> K	Q: neutral<br>K: + charge	all tools:<br>neutral (83%)	DDG: -0.47,<br>RI: 3	other species:<br>various amino acids<br>CS: 6
		154	TTT -> F	CTT -> L	F: non-polar, no S-group<br>L: neutral, hydrophobic side chain	MAPP tool:<br>deleterious (51%)<br>PolyPhen-1 tool:<br>deleterious (59%)<br>PolyPhen-2 tool:<br>deleterious (50%)<br>other tools:<br>neutral (60%)	DDG: -1.05,<br>RI: 5	other species:<br>various amino acids<br>CS: 4
		160	ACT -> T	TCT -> S	T/S: polar	all tools:<br>neutral (83%)	DDG: -0.25,<br>RI: 3	other species:<br>various amino acids<br>CS: 4
		181	ATC -> I	TTA -> L	I/L: neutral, hydrophobic side chain	PolyPhen-2 tool:<br>deleterious (40%)<br>other tools:<br>neutral (75%)	DDG: -0.96,<br>RI: 5	other species:<br>various amino acids<br>CS: 3
		193	AGG -> T	ATC -> I	T: polar	MAPP tool:<br>deleterious (48%)	DDG: -1.80,<br>RI: 8	other species:<br>various amino acids

					I: neutral, hydrophobic side chain	other tools: neutral (74%)		CS: 5
		199	GGC -> G	GCC -> A	G: non-polar, no S-group<br>A: hydrophobic side chain, non-polar	MAPP tool: deleterious (57%)<br>other tools: neutral (74%)	DDG: -1.11, RI: 8	other species: various amino acids, but never G<br>CS: 5
		226	CTC -> L	ACT -> T	L: neutral, hydrophobic side chain<br>T: polar	all tools: neutral (83%)	DDG: -0.09, RI: 2	other species: various amino acids<br>CS: 1
		229	ATA -> I	TAT -> Y	I: neutral, hydrophobic side chain<br>Y: polar	MAPP tool: deleterious (57%)<br>other tools: neutral (74%)	DDG: 0.02, RI: 3	other species: various amino acids<br>CS: 1
		259	AAT -> N	AGC -> S	N: neutral, polar<br>S: polar	Deleterious (61%) in all tools, except for PhD-SNP (neutral 68%) and PolyPhen-1 (neutral 67%)	DDG: -0.15, RI: 5	other species: A, S<br>CS: 7
		298	TAT -> Y	TTT -> F	Y: polar<br>F: non-polar, no S-group	all tools: neutral (83%)	DDG: -0.54, RI: 1	other species: various amino acids<br>CS: 5
		307	GCC -> A	GAA -> E	A: hydrophobic side chain, non-polar<br>E: polar, + charge	MAPP tool: deleterious (84%)<br>SIFT tool: deleterious (43%)<br>other tools: neutral (63%)	DDG: -0.40, RI: 9	other species: various amino acids<br>CS: 5
		322	AAA -> K	ACG -> T	K: + charge<br>T: polar	all tools: neutral (83%)	DDG: -0.65, RI: 4	other species: various amino acids<br>CS: 1

		325	CAA -> Q	CGT -> R	Q: neutral<br>R: + charge	PolyPhen-2 tool: deleterious (43%)<br>other tools: neutral (74%)	DDG: -0.59,<br>RI: 6	other species: various amino acids<br>CS: 5
		331	ATC -> I	ACC -> T	I: neutral, hydrophobic side chain<br>T: polar	MAPP tool: deleterious (51%)<br>other tools: neutral (74%)	DDG: -0.07,<br>RI: 1	other species: various amino acids<br>CS: 1
		367	GCC -> A	GAA -> E	A: hydrophobic side chain, non-polar<br>E: polar, + charge	Deleterious (51%) in all tools, except for PhD-SNP (neutral 78%) and PolyPhen-1 (neutral 67%)	DDG: -0.34,<br>RI: 6	other species: various amino acids<br>CS: 8
		370	TTA -> L	ATG -> M	L: neutral, hydrophobic side chain<br>M: non-polar	MAPP tool: deleterious (43%)<br>other tools: neutral (74%)	DDG: -0.61,<br>RI: 6	other species: M, F, L, I, V<br>CS: 7
		382	ATT -> I	GTT -> V	I: neutral, hydrophobic side chain<br>V: neutral, hydrophobic	MAPP tool: deleterious (77%)<br>PolyPhen-2 tool: deleterious (43%)<br>SIFT tool: deleterious (79%)<br>other tools: neutral (60%)	DDG: -0.30,<br>RI: 5	other species: M, V, F, I, L<br>CS: 6
		406	GGT -> G	GCC -> A	G: non-polar, no S-group<br>A: hydrophobic side chain, non-polar	PolyPhen-2 tool: deleterious (43%)<br>other tools: neutral (74%)	DDG: -1.14,<br>RI: 6	other species: A, T, G, S<br>CS: 7
	Ribosomal silencing factor RsfS	25	ATG -> M	CTA -> L	M: non-polar	Deleterious (52%) in all tools, except for PhD-SNP tool	DDG: -1.01,<br>RI: 9	other species: various amino acids<br>CS: 3

					L: neutral, hydrophobic side chain	(neutral 83%) and SNAP tool (neutral 67%)		
		49	AAT -> N	GAC -> D	N: neutral, polar<br>D: + charge	PolyPhen-2 tool: deleterious (43%)<br>other tools: neutral (74%)	DDG: 0.03,<br>RI: 5	other species: L, F, D, A, E, N, S, G<br>CS: 5
		52	GTC -> V	ATC -> I	V: neutral, hydrophobic<br>I: neutral, hydrophobic side chain	MAPP tool: deleterious (51%)<br>other tools: neutral (74%)	DDG: -0.62,<br>RI: 6	other species: I, L, V<br>CS: 8
		112	TCC -> S	ACC -> T	S: polar<br>T: polar	all tools: neutral (83%)	DDG: -0.67,<br>RI: 8	other species: V, G, T, A, S<br>CS: 7
		181	GTG -> V	GAG -> E	V: neutral, hydrophobic<br>E: polar, + charge	PolyPhen-2 tool: deleterious (43%)<br>other tools: neutral (74%)	DDG: 0.51,<br>RI: 3	other species: various amino acids<br>CS: 1
		193	GTG -> V	ATA-> I	V: neutral, hydrophobic<br>I: neutral, hydrophobic side chain	all tools:<br>neutral (83%)	DDG: -0.83,<br>RI: 7	other species: various amino acids<br>CS: 4
		199	GAG -> E	GAT -> D	E: polar, + charge<br>D: + charge	all tools:<br>neutral (83%)	DDG: 0.11,<br>RI: 5	other species: I, G, R, T, D, K, A, E<br>CS: 7
		202	AGC -> S	GGC -> G	S: polar<br>G: non-polar, no S-group	all tools:<br>neutral (83%)	DDG: -0.99,<br>RI: 8	other species: various amino acids<br>CS: 3
		220	ATT-> I	GTT -> V	I: neutral, hydrophobic side chain	all tools:<br>neutral (83%)	DDG: -0.04,<br>RI: 1	other species: I, L, A, V<br>CS: 7

					V: neutral, hydrophobic			
		244	GTG -> V	ATT -> I	V: neutral, hydrophobic<br>I: neutral, hydrophobic side chain	MAPP tool: deleterious (48%)<br>other tools: neutral (74%)	DDG: -0.90, RI: 8	other species: V, I, L<br>CS: 7
		250	ATT -> I	CTT -> L	I/L: neutral, hydrophobic side chain	all tools: neutral (83%)	DDG: -1.23, RI: 7	other species: L, I, M, V<br>CS: 7
		271	GAA -> E	GAC -> D	E: polar, + charge<br>D: + charge	all tools: neutral (83%)	DDG: -0.34, RI: 0	other species: various amino acids<br>CS: 1
		307	CAC -> H	AAT -> N	H: + charge<br>N: neutral, polar	all tools: neutral (83%)	DDG: -0.47, RI: 7	other species: various amino acids<br>CS: 1
		316	CAT -> H	AAT -> N	H: + charge<br>N: neutral, polar	PolyPhen-1 tool: deleterious (74%)<br>PolyPhen-2 tool: deleterious (55%)<br>SIFT tool: deleterious (43%)<br>other tools: neutral (60%)	DDG: -0.34, RI: 6	other species: N, H<br>CS: 6
	Ribosomal RNA small subunit methyltransferase D (RsmD)	124	AGC -> S<br>AAC -> N	GAC -> D	S: polar<br>N: neutral, polar<br>D: + charge	all tools: neutral (83%)	DDG: -0.53, RI: 2<br>DDG: -0.89, RI: 2	other species: various amino acids<br>CS: 1
		205	TTT -> F<br>CAC -> H	TAT -> Y	F: non-polar, no S-group<br>H: + charge<br>Y: polar	all tools: neutral (83%)	DDG: -0.12, RI: 1<br>DDG: -1.06, RI: 5	other species: various amino acids<br>CS: 4
		217	GTG -> V<br>ATT -> I	TTT -> F	V/I: neutral, hydrophobic	all tools: deleterious (87%)	DDG: -0.63, RI: 5	other species: various amino acids

					F: non-polar, no S-group			CS: 8
		232	GAA -> Q<br>AAA -> K	GCG -> A	Q: neutral<br>A: hydrophobic side chain, non-polar	PolyPhen-1 tool: deleterious (59%)<br>PolyPhen-2 tool: deleterious (54%)<br>other tools: neutral (63%)	DDG: -0.46, RI: 6	other species: various amino acids<br>CS: 3
		235	GAA -> E	CAG -> Q	E: polar, + charge<br>Q: neutral	all tools: neutral (83%)	DDG: 0.01, RI: 4	other species: various amino acids<br>CS: 1
		244	GGG -> G<br>TTG -> L	AAG -> K	G: non-polar, no S-group<br>L: neutral, hydrophobic side chain<br>K: + charge	all tools: neutral (83%)	DDG: -0.51, RI: 4<br><br>DDG: 0.72, RI: 7	other species: various amino acids<br>CS: 2
		271	TTT -> F<br>TTA -> L	ATC -> I	F: non-polar, no S-group<br>L/I: neutral, hydrophobic side chain	PhD-SNP tool: deleterious (59%)<br>other tools: neutral (75%)	DDG: -1.01, RI: 5<br><br>DDG: -0.42, RI: 4	other species: C, A, L, I, Y, T, V, F<br>CS: 5
		289	ACC -> T	CAA -> Q	T: polar<br>Q: neutral	PolyPhen-2 tool: deleterious (40%)<br>other tools: neutral (74%)	DDG: -0.22, RI:6	other species: various amino acids<br>CS: 1
		301	AGA -> R	AAG -> K	R/K: + charge	MAPP tool: deleterious (48%)<br>other tools: neutral (74%)	DDG: 0.03, RI: 2	other species: various amino acids<br>CS: 2
		310	CAC -> H	TGC -> C	H: + charge<br>C: polar, S-group	MAPP tool: deleterious (76%)	DDG: -0.91, RI: 6	other species: various amino acids

						other tools:<br>neutral (75%)		CS: 1
		319	ATA > I	GTG -> V	I: neutral, hydrophobic side chain<br>V: neutral, hydrophobic	SIFT tool:<br>deleterious (53%)<br>other tools:<br>neutral (74%)	DDG: -0.41,<br>RI: 6	other species:<br>various amino acids<br>CS: 7
		322	TTT -> F	TTG -> L	F: non-polar, no S-group<br>L: neutral, hydrophobic side chain	all tools:<br>neutral (83%)	DDG: -1.09,<br>RI: 8	other species: A<br>various amino acids<br>CS: 7
		328	ACC -> T	GCG -> A	T: polar<br>A: hydrophobic side chain, non-polar	MAPP tool:<br>deleterious (51%)<br>PolyPhen-2 tool:<br>deleterious (43%)<br>other tools:<br>neutral (68%)	DDG: -0.45,<br>RI: 2	other species:<br>various amino acids<br>CS: 2
		331	CTA -> L	TTT -> F	L: neutral, hydrophobic side chain<br>F: non-polar, no S-group	all tools:<br>neutral (83%)	DDG: -1.04,<br>RI: 7	other species:<br>various amino acids<br>CS: 7
		334	GCG -> A	AGC -> S	A: hydrophobic side chain, non-polar<br>S: polar	all tools:<br>neutral (83%)	DDG: -0.60,<br>RI: 9	other species:<br>various amino acids<br>CS: 1
		340	TAT -> Y	CAT -> H	Y: polar<br>H: + charge	MAPP tool:<br>deleterious (41%)<br>other tools:<br>neutral (75%)	DDG: 0.31,<br>RI: 7	other species:<br>various amino acids<br>CS: 4
		346	GAA -> E	AAG -> K	E: polar, + charge<br>K: + charge	PhD-SNP tool:<br>deleterious (59%)<br>other tools:	DDG: 0.02,<br>RI: 3	other species:<br>various amino acids<br>CS: 1

						neutral (75%)		
		355	AAG -> K	GAG -> E	K: + charge<br>E: polar, + charge	all tools:<br>neutral (83%)	DDG: -0.79,<br>RI: 8	other species:<br>various amino acids<br>CS: 1
		430	GTG -> V	CTA -> L	V: neutral, hydrophobic<br>L: neutral, hydrophobic side chain	Deleterious (51%) in all tools, except for PolyPhen-1 tool (neutral 67%), SIFT tool (neutral 68%) and SNAP tool (neutral 61%)	DDG: -1.48,<br>RI: 8	other species: M, V, L, I, F<br>CS: 7
		433	GCA -> A	GAA -> E	A: hydrophobic side chain, non-polar<br>E: polar, + charge	MAPP tool:<br>deleterious (63%)<br>other tools:<br>neutral (75%)	DDG: -0.23,<br>RI: 4	other species:<br>various amino acids<br>CS: 3
		442	AGG -> R	AAT -> N	R: + charge<br>N: neutral, polar	all tools:<br>neutral (83%)	DDG: 0.39,<br>RI: 4	other species:<br>various amino acids<br>CS: 2
		445	GGT > G	CCT -> P	G: non-polar, no S-group<br>P: aromatic side chain	all tools:<br>neutral (83%)	DDG: -1.62,<br>RI: 9	other species: G, P, I<br>CS: 1
		463	CAA -> Q	AAG -> K	Q: neutral<br>K: + charge	PolyPhen-2 tool:<br>deleterious (43%)<br>other tools:<br>neutral (74%)	DDG: -0.26,<br>RI: 2	other species:<br>various amino acids<br>CS: 1
		466	TAT -> Y	CAT -> H	Y: polar<br>H: + charge	PolyPhen-1 tool:<br>deleterious (59%)<br>PolyPhen-2 tool:<br>deleterious (50%)<br>other tools:<br>neutral (74%)	DDG: 0.50,<br>RI: 8	other species:<br>various amino acids<br>CS: 1

		472	CTT -> L	TTT -> F	L: neutral, hydrophobic side chain<br>F: non-polar, no S-group	all tools:<br>neutral (83%)	DDG: -1.04,<br>RI: 8	other species:<br>various amino acids<br>CS: 1
		505	GTG -> C	AGT -> S	C: polar, S-group<br>S: polar	PolyPhen-1 tool:<br>deleterious (74%)<br>PolyPhen-2 tool:<br>deleterious (81%)<br>SIFT tool:<br>deleterious (46%)<br>other tools:<br>neutral (60%)	DDG: -0.50,<br>RI: 3	other species:<br>various amino acids<br>CS: 2
		514	AAG -> K	AAT -> N	K: + charge<br>N: neutral, polar	PolyPhen-2 tool:<br>deleterious (41%)<br>other tools:<br>neutral (74%)	DDG: -0.27,<br>RI: 2	other species:<br>various amino acids<br>CS: 1
		517	ACG -> T	GCG -> A	T: polar<br>A: hydrophobic side chain, non-polar	MAPP tool:<br>deleterious (51%)<br>PolyPhen-2 tool:<br>deleterious (40%)<br>SIFT tool:<br>deleterious (45%)<br>other tools:<br>neutral (60%)	DDG: -0.74,<br>RI: 6	other species:<br>various amino acids<br>CS: 5
		524	ACA -> T	GAA -> E	T: polar<br>E: polar, + charge	PolyPhen-2 tool:<br>deleterious (43%)<br>other tools:<br>neutral (74%)	DDG: -0.38,<br>RI: 2	other species:<br>various amino acids<br>CS: 5
		527	ACA > T	AAG -> K	T: polar<br>K: + charge	Deleterious (51%) in all tools, except for PhD-SNP tool (neutral 72%), PolyPhen-1 tool	DDG: -0.62,<br>RI: 5	other species:<br>various amino acids<br>CS: 8

						(neutral 67%), and SNAP tool (neutral 50%)		
	Ribosomal RNA small subunit methyltransferase I (RsmI)	73	AAC -> N<br>AGC ->S	GGC -> G	N: neutral, polar<br>S: polar<br>G: non-polar, no S-group	all tools:<br>neutral (83%)	DDG: -0.76, RI: 6<br>DDG: -0.84, RI: 8	other species: various amino acids<br>CS: 2
		79	GGC -> A<br>GAT -> D	GGT -> G	A: hydrophobic side chain, non-polar<br>D: + charge<br>G: non-polar, no S-group	**G27A** -> MAPP tool: deleterious (48%) other tools: neutral (74%)<br>**G27D** -> all tools: neutral (83%)	DDG: -0.57, RI: 8<br>DDG: -0.89, RI: 8	other species: various amino acids<br>CS: 7
		136	AGT -> Q<br>CAA -> S	GAG -> E	Q: neutral<br>S: polar<br>E: polar, + charge	all tools:<br>neutral (83%)	DDG: 0.02, RI: 0<br>DDG: -0.29, RI:	other species: various amino acids<br>CS: 7
		169	GCC-> A<br>CAA -> Q	AAA -> K	A: hydrophobic side chain, non-polar<br>Q: neutral<br>K: + charge	all tools:<br>neutral (83%)	DDG: -0.22, RI: 6<br>DDG: -0.16, RI: 0	other species: various amino acids<br>CS: 4
		178	CAG -> Q	TGT -> C	Q: neutral<br>C: polar. S-group	all tools:<br>neutral (83%)	DDG: -0.46, RI: 4	other species: various amino acids<br>CS: 4
		211	GCG -> A	ACC -> T	T: polar<br>A: hydrophobic side chain, non-polar	all tools:<br>neutral (83%)	DDG: -0.81, RI: 5	other species: various amino acids<br>CS: 1
		334	AAC -> N<br>AAG -> K	AGC -> S	N: neutral, polar<br>K: + charge<br>S: polar	**S112N** -> all tools: neutral (83%)<br>**S112K** -> PhD-SNP tool: deleterious (73%) other tools: neutral (75%)	DDG: -0.19, RI: 3<br>DDG: -0.17, RI: 0	other species: various amino acids<br>CS: 3

		445	CTA -> L<br>CAA -> Q	AAG -> K	L: neutral, hydrophobic side chain<br>Q: neutral<br>K: + charge	**K149L** -> MAPP tool: deleterious (43%), PhD-SNP tool: deleterious (59%), PANTHER tool: deleterious (57%) other tools: neutral (65%)<br>**K149Q** -> PhD-SNP tool: deleterious (58%) other tools: neutral (74%)	DDG: 0.36, RI: 1<br>DDG: -0.10, RI: 0	other species: V, A, G, T, Q, R, N, S, K but never L<br>CS: 4
		454	CGC -> R	TGC -> C	R: + charge<br>C: polar, S-group	PANTHER tool: deleterious (74%) other tools: neutral (83%)	DDG: 0.07, RI: 2	other species: various amino acids<br>CS: 4
		484	GGG -> G	ACG -> T	A: hydrophobic side chain, non-polar<br>T: polar	all tools: neutral (83%)	DDG: -1.50, RI: 9	other species: various amino acids<br>CS: 1
		535	GAA -> E	GCA -> A	E: polar, + charge<br>A: hydrophobic side chain, non-polar	PhD-SNP tool: deleterious (59%) other tools: neutral (74%)	DDG: 0.0, RI: 4	other species various amino acids<br>CS: 1
		565	AGT -> S	TAT -> Y	S: polar<br>Y: polar	all tools: neutral (83%)	DDG: -1.05, RI: 5	other species: various amino acids<br>CS: 1
		577	ATA -> I	ACA -> T	I: neutral, hydrophobic side chain<br>T: polar	all tools: neutral (83%)	DDG: -0.08, RI: 2	other species: various amino acids<br>CS: 6
		625	CTT -> L	CCA -> P	L: neutral, hydrophobic side chain	all tools: neutral (83%)	DDG: -0.02, RI: 1	other species: various amino acids<br>CS: 1

					P: aromatic side chain			
			-> E<br>-> K	-> Q	E: - charge<br>K: + charge<br>Q: neutral	**Q210E** -> all tools: neutral (83%)<br>**Q210K** -> SIFT tool: deleterious (46%), other tools: neutral (74%)	DDG: 0.10, RI: 4<br><br>DDG: -0.23, RI: 1	other species: various amino acids<br>CS: 6
		637	ACA -> T	GCC -> A	T: polar<br>A: hydrophobic side chain, non-polar	PolyPhen-2 tool: deleterious (40%) other tools: neutral (74%)	DDG: -0.36, RI: 4	other species: various amino acids<br>CS: 2
		655	CAG -> Q	CAC -> H	Q: neutral<br>H: + charge	all tools: neutral (83%)	DDG: 0.26, RI: 1	other species: various amino acids<br>CS: 1
			-> L<br>-> A	-> P	L: neutral, hydrophobic side chain<br>A: hydrophobic side chain, non-polar<br>P: aromatic side chain	**P240L** -> all tools: neutral (83%)<br>**P240A** -> PolyPhen-2 tool: deleterious (40%), other tools: neutral (75%)	DDG: -0.01, RI: 1<br><br>DDG: -1.01, RI:6	other species: various amino acids<br>CS: 1
		742	AGC -> S	ATG -> M	S: polar<br>M: non-polar	MAPP tool: deleterious (41%) other tools: neutral (74%)	DDG: -0.96, RI: 3	other species: various amino acids<br>CS: 7
		796	AAC -> N	CAC -> H	N: neutral, polar<br>H: + charge	all tools: neutral (83%)	DDG: -0.12, RI: 1	other species: N, E, D, G, Q, H<br>CS: 5
	Ribosomal RNA small subunit methyltransferase H (RsmH)	37	CTT -> L	TTT -> F	L: neutral, hydrophobic side chain<br>F: non-polar, no S-group	all tools: neutral (83%)	DDG: -0.89, RI: 8	other species: L, F<br>CS: 3

		43	AAA -> K	GAG -> E	K: + charge<br>E: polar, + charge	all tools:<br>neutral (83%)	DDG: -0.68,<br>RI: 8	other species:<br>various amino acids<br>CS: 4
		58	AGG -> R	AAG -> K	R/K: + charge	all tools:<br>neutral (83%)	DDG: 0.04,<br>RI: 1	other species:<br>various amino acids<br>CS: 3
		70	ATT -> I	GTT -> V	I: neutral,<br>hydrophobic side chain<br>V: neutral,<br>hydrophobic	all tools:<br>neutral (83%)	DDG: -0.33,<br>RI: 4	other species:<br>various amino acids<br>CS: 3
		76	ATT -> I	GTG -> V	I: neutral,<br>hydrophobic side chain<br>V: neutral,<br>hydrophobic	all tools:<br>neutral (83%)	DDG: -0.36,<br>RI: 4	other species: C, V, A, M, L, I, F<br>CS: 6
		121	CAA -> Q	GAA -> E	Q: neutral<br>E: polar, + charge	all tools:<br>neutral (83%)	DDG: -0.41,<br>RI: 5	other species:<br>various amino acids<br>CS: 1
		133	AAT -> N	CGC -> R	L: neutral,<br>hydrophobic side chain<br>F: non-polar, no S-group	all tools:<br>neutral (83%)	DDG: -1.12,<br>RI: 8	other species:<br>various amino acids<br>CS: 1
		139	CAC -> H	CGT -> R	H/R: + charge	PANTHER tool:<br>deleterious (69%)<br>other tools:<br>neutral (83%)	DDG: -1.23,<br>RI: 9	other species:<br>various amino acids<br>CS: 4
		142	ATT -> I	GTC -> V	I: neutral,<br>hydrophobic side chain<br>V: neutral,<br>hydrophobic	all tools:<br>neutral (83%)	DDG: -0.32,<br>RI: 4	other species: F, I, L, Y, A, V<br>CS: 7

		145	GTC > V	ATA -> I	V: neutral, hydrophobic<br>I: neutral, hydrophobic side chain	all tools:<br>neutral (83%)	DDG: -1.04,<br>RI: 8	other species:<br>various amino acids<br>CS: 5
		163	AAA -> K	CGC -> R	K/R: + charge	all tools:<br>neutral (83%)	DDG: -0.92,<br>RI: 8	other species:<br>various amino acids<br>CS: 1
		166	GAA -> E	CAA -> Q	E: polar, + charge<br>Q: neutral	all tools:<br>neutral (83%)	DDG: -0.32,<br>RI: 4	other species:<br>various amino acids<br>CS: 1
		172	CAA -> Q	AGC -> S	Q: neutral<br>S: polar	PhD-SNP tool:<br>deleterious (61%)<br>other tools:<br>neutral (74%)	DDG: -0.12,<br>RI: 3	other species:<br>various amino acids<br>CS: 4
		184	CTT -> L	TCA -> S	L: neutral, hydrophobic side chain<br>S: polar	PhD-SNP tool:<br>deleterious (61%)<br>other tools:<br>neutral (74%)	DDG: 0.41,<br>RI: 7	other species:<br>various amino acids<br>CS: 1
		187	GAC -> D	GAA -> E	D: + charge<br>E: polar, + charge	PhD-SNP tool:<br>deleterious (61%)<br>SIFT tool:<br>deleterious (45%)<br>other tools:<br>neutral (65%)	DDG: -0.61,<br>RI: 5	other species:<br>various amino acids<br>CS: 2
		190	AAA -> K	AGA -> R	K/R: + charge	PhD-SNP tool:<br>deleterious (59%)<br>other tools:<br>neutral (74%)	DDG: -0.71,<br>RI: 6	other species:<br>various amino acids<br>CS: 5
		196	ACC -> T	GCC > A	T: polar<br>A: hydrophobic side chain, non-polar	MAPP tool:<br>deleterious (46%)<br>PhD-SNP tool:<br>deleterious (77%)<br>other tools:	DDG: -0.83,<br>RI: 8	other species:<br>various amino acids<br>CS: 1

					neutral (63%)		
		199	CCC -> P	GCC -> A	P: aromatic side chain<br>A: hydrophobic side chain, non-polar	PhD-SNP tool: deleterious (61%)<br>other tools: neutral (74%)	DDG: -0.09, RI: 3	other species: various amino acids<br>CS: 1
		205	AAA -> K	ACG -> T	K: + charge<br>T. polar	all tools: neutral (83%)	DDG: -1.05, RI: 8	other species: various amino acids<br>CS: 1
		209	GAG -> E	GAC -> D	E: polar, + charge<br>D: + charge	all tools: neutral (83%)	DDG: 0.01, RI: 0	other species: various amino acids<br>CS: 1
		217	ACC -> T	AAC -> N	T: polar<br>N: neutral, polar	all tools: neutral (83%)	DDG: -0.23, RI: 3	other species: various amino acids<br>CS: 1
		220	CAC -> H	TAT -> Y	H: + charge<br>Y: polar	MAPP tool: deleterious (76%)<br>PhD-SNP tool: deleterious (59%)<br>other tools: neutral (63%)	DDG: -1.32, RI: 4	other species: various amino acids<br>CS: 3
		223	AAA -> K	TGT -> C	K: + charge<br>C: polar, S-group	PhD-SNP tool: deleterious (61%)<br>other tools: neutral (75%)	DDG: -0.76, RI: 6	other species: various amino acids<br>CS: 2
		227	CAG -> Q	GCG -> A	Q: neutral<br>A: hydrophobic side chain, non-polar	PhD-SNP tool: deleterious (68%)<br>other tools: neutral (75%)	DDG: -0.67, RI: 8	other species: various amino acids<br>CS: 3
		241	CAA -> Q	ACA -> T	Q: neutral<br>T: polar	MAPP tool: deleterious (59%)<br>other tools: neutral (74%)	DDG: -0.99, RI: 7	other species: various amino acids<br>CS: 1
		262	TCC -> S	CCC -> P	S: polar	PhD-SNP tool: deleterious (59%)	DDG: -1.29, RI: 8	other species: various amino acids

					P: aromatic side chain	PANTHER tool: deleterious (70%) other tools: neutral (74%)		CS: 1
		265	CCA -> P	ATT -> I	P: aromatic side chain I: neutral, hydrophobic side chain	Deleterious (51%) in all tools, except for PolyPhen-1 tool (neutral 67%), PolyPhen-2 tool (neutral 69%) and SNAP tool (neutral 61%)	DDG: -1.60, RI: 6	other species: various amino acids CS: 6
		268	CAG -> Q	GAT -> D	Q: neutral D: + charge	MAPP tool: deleterious (46%) PhD-SNP tool: deleterious (86%) PolyPhen-2 tool: deleterious (43%) PANTHER tool: deleterious (57%) other tools: neutral (60%)	DDG: -0.36, RI: 3	other species: various amino acids CS: 7
		313	AAC -> N	AGC -> S	N: neutral, polar S: polar	all tools: neutral (83%)	DDG: -0.47, RI: 2	other species: various amino acids CS: 1
		319	AAT -> N	CAT -> H	N: neutral, polar H: + charge	all tools: neutral (83%)	DDG: -0.54, RI: 1	other species: various amino acids CS: 4
		337	AAC -> N	CAC -> H	N: neutral, polar H: + charge	all tools: neutral (83%)	DDG: -0.67, RI: 2	other species: various amino acids CS: 4
		340	GCC -> A	TCT -> S	A: hydrophobic side chain, non-polar S: polar	MAPP tool: deleterious (62%) SIFT tool:	DDG: -0.77, RI: 8	other species: various amino acids CS: 7

						deleterious (45%)<br>other tools:<br>neutral (65%)		
		346	AGC -> S	AAT -> N	S: polar<br>N: neutral, polar	all tools:<br>neutral (83%)	DDG: -0.45,<br>RI: 4	other species:<br>various amino acids<br>CS: 6
		352	ACG -> T	ATG -> M	T: polar<br>M: non-polar	PANTHER tool:<br>deleterious (61%)<br>other tools:<br>neutral (83%)	DDG: -1.09,<br>RI: 7	other species:<br>various amino acids<br>CS: 1
		355	GGG -> G	GAG -> E	G: non-polar, no S-group<br>E: polar, + charge	all tools:<br>neutral (83%)	DDG: -1.09,<br>RI: 6	other species:<br>various amino acids<br>CS: 1
		403	GGC -> G	CAT -> H	G: non-polar, no S-group<br>H: + charge	PhD-SNP tool:<br>deleterious (58%)<br>other tools:<br>neutral (74%)	DDG: -0.68,<br>RI: 3	other species:<br>various amino acids<br>CS: 1
		439	ACG -> T	GCG -> A	T: polar<br>A: hydrophobic side chain, non-polar	all tools:<br>neutral (83%)	DDG: -0.52,<br>RI: 4	other species:<br>various amino acids<br>CS: 6
		484	CAA -> Q	CGG -> R	Q: neutral<br>R: + charge	all tools:<br>neutral (83%)	DDG: -0.92,<br>RI: 8	other species:<br>various amino acids<br>CS: 1

Supplementary Table S6. Genes investigated for the antimicrobial resistance mechanism with no SNPs founded.

	**Gene**	**Protein**	**SNPs**
Acquired resistance to azithromycin (macrolide) in ASB19	*rplA*	50S ribosomal protein L1	No
	rplJ	50S ribosomal protein L10	No
	rplK	50S ribosomal protein L11	No
	rplM	50S ribosomal protein L13	No
	rplN	50S ribosomal protein L14	No
	rplO	50S ribosomal protein L15	No
	rplP	50S ribosomal protein L16	No
	rplQ	50S ribosomal protein L17	No
	rplR	50S ribosomal protein L18	No
	rplS	50S ribosomal protein L19	No
	rplT	50S ribosomal protein L20	No
	rplU	50S ribosomal protein L21	No
	rplV	50S ribosomal protein L22	No
	rplW	50S ribosomal protein L23	No
	rplX	50S ribosomal protein L24	No
	rplY	50S ribosomal protein L25	No
	rpmA	50S ribosomal protein L27	No
	rpmB	50S ribosomal protein L28	No
	rpmC	50S ribosomal protein L29	No
	rpmE	50S ribosomal protein L31	No
	rpmF	50S ribosomal protein L32	No
	rpmG2	50S ribosomal protein L33 2	No
	rpmH	50S ribosomal protein L34	No
	group_744	50S ribosomal protein L35	No
	rplD	50S ribosomal protein L4	No
	rplE	50S ribosomal protein L5	No
	rplF	50S ribosomal protein L6	No
	rplL	50S ribosomal protein L7/L12	No
	rplI	50S ribosomal protein L9	No
	rluA	Ribosomal large subunit pseudouridine synthase A	No

	rluB	Ribosomal large subunit pseudouridine synthase B	No
	rluD_2	Ribosomal large subunit pseudouridine synthase D	No
	rluD_1	Ribosomal large subunit pseudouridine synthase D	No
	prmA	Ribosomal protein L11 methyltransferase	No
Acquired resistance to spectinomycin (aminoglycoside) in ASB1	*rpsJ*	30S ribosomal protein S10	No
	rpsK	30S ribosomal protein S11	No
	rpsM	30S ribosomal protein S13	No
	rpsZ	30S ribosomal protein S14 type Z	No
	rpsO	30S ribosomal protein S15	No
	rpsP	30S ribosomal protein S16	No
	rpsQ	30S ribosomal protein S17	No
	rpsR	30S ribosomal protein S18	No
	rpsS	30S ribosomal protein S19	No
	rpsB	30S ribosomal protein S2	No
	rpsT	30S ribosomal protein S20	No
	rpsU	30S ribosomal protein S21	No
	rpsC	30S ribosomal protein S3	No
	rpsD	30S ribosomal protein S4	No
	rpsE	30S ribosomal protein S5	No
	rpsF	30S ribosomal protein S6	No
	rpsH	30S ribosomal protein S8	No
	rpsI	30S ribosomal protein S9	No
	rimO	Ribosomal protein S12 methylthiotransferase RimO	No
	rlmH group_1616	Ribosomal RNA large subunit methyltransferase H	No
	rsmA group_2011	Ribosomal RNA small subunit methyltransferase A	No
	rsmE group_892	Ribosomal RNA small subunit methyltransferase E	No
	rsmG	Ribosomal RNA small subunit methyltransferase G	No
	rsmH	Ribosomal RNA small subunit methyltransferase H	No
	rsmI	Ribosomal RNA small subunit methyltransferase I	No
	rsfS	Ribosomal silencing factor RsfS	No
	rpsJ	30S ribosomal protein S10	No
	rpsK	30S ribosomal protein S11	No

Aminoglycoside decreased susceptibility in *H. heilmannii* isolates	*rpsL*	30S ribosomal protein S12	No
	rpsM	30S ribosomal protein S13	No
	rpsZ	30S ribosomal protein S14 type Z	No
	rpsQ	30S ribosomal protein S17	No
	rpsR	30S ribosomal protein S18	No
	rpsS	30S ribosomal protein S19	No
	rpsT	30S ribosomal protein S20	No
	rpsC	30S ribosomal protein S3	No
	rpsD	30S ribosomal protein S4	No
	rpsE	30S ribosomal protein S5	No
	rpsG	30S ribosomal protein S7	No
	rpsH	30S ribosomal protein S8	No
	rsmE group_892	Ribosomal RNA small subunit methyltransferase E	No
	rsmG	Ribosomal RNA small subunit methyltransferase G	No
	rsmA group_2011	Ribosomal RNA small subunit methyltransferase A	No

Supplementary Table S7. Comparison of MIC values of *H. heilmannii* and *H. ailurogastricus* with others gastric *Helicobacter* spp.

	**MICs *H. heilmannii* (µg/ml)**	**MICs *H. ailurogastricus* (µg/ml)**	**MICs other gastric *Helicobacter* sp. (µg/ml)**	**References**
**Ampicillin**	0.125 - 1	0.125 - 1	*H. pylori*: 0.015-0.125<br>*H. suis*: 0.125-8<br>*H. bizzozeronii*: 0.06-0.125<br>*H. felis*: 0.03-0.25<br>*H. salomonis*: 0.03-0.125	Loo *et al*., 1997<br>Van den Bulck *et al*., 2005<br>Berlamont *et al.*, 2019
**Ceftiofur**	0.5 - 8	4 - 16	*H. pylori*: no data<br>*H. suis*: 1-32<br>*H. bizzozeronii*: no data<br>*H. felis*: no data<br>*H. salomonis*: no data	Berlamont *et al.*, 2019
**Clarithromycin**	≤0.03125 - 0.25	0.0625 – 0.25	*H. pylori*: 0.015-0.125<br>*H. suis*: 0.03-0.5<br>*H. bizzozeronii*: 0.06-0.125<br>*H. felis*: 0.03-0.25<br>*H. salomonis*: 0.03	Van den Bulck *et al*., 2005<br>Berlamont *et al.*, 2019
**Tylosin**	0.5 - 8	1 - 4	*H. pylori*: no data<br>*H. suis*: 0.5-16<br>*H. bizzozeronii*: 0.125-0.5<br>*H. felis*: 0.03-0.25<br>*H. salomonis*:0.03-0.125	Van den Bulck *et al*., 2005<br>Berlamont *et al.*, 2019
**Azithromycin**	≤0.03125 - 0.25	≤0.03125 - 0.125	*H. pylori*: no data<br>*H. suis*: no data<br>*H. bizzozeronii*: no data<br>*H. felis*: no data<br>*H. salomonis*: no data	
**Lincomycin**	0.5 - 32	4 - 16	*H. pylori*: no data<br>*H. suis*: 2-128<br>*H. bizzozeronii*: 0.5-8<br>*H. felis*: 0.25-8<br>*H. salomonis*: 0.25-1	Van den Bulck *et al*., 2005<br>Berlamont *et al.*, 2019
**Enrofloxacin**	≤0.03125 - 0.25	≤0.03125 - 0.25	*H. pylori*: no data	Van den Bulck *et al*., 2005

			H. suis: 0.03-8<br>*H. bizzozeronii*: 0.03-0.125<br>*H. felis*: 0.03-0.06<br>*H. salomonis*: 0.03-0.125	Berlamont *et al.*, 2019
**Levofloxacin**	≤0.03125 - 0.25	≤0.03125 - 0.0625	*H. pylori*: no data<br>*H. suis*: 0.03-32<br>*H. bizzozeronii*: no data<br>*H. felis*: no data<br>*H. salomonis*: no data	Berlamont *et al.*, 2019
**Rifampicin**	≤0.03125 - 0.125	≤0.03125 - 0.25	*H. pylori*: no data<br>*H. suis*: 0.03-4<br>*H. bizzozeronii*: no data<br>*H. felis*: no data<br>*H. salomonis*: no data	Berlamont *et al.*, 2019
**Gentamicin**	2 - 32	4 - 16	*H. pylori*: no data<br>*H. suis*: 1-16<br>*H. bizzozeronii*: 0.06-0.25<br>*H. felis*: 0.06-0.25<br>*H. salomonis*: 0.125	Van den Bulck *et al.*, 2005<br>Berlamont *et al.*, 2019
**Spectinomycin**	≤0.03125 - 32	0.0625 - 0.25	*H. pylori*: no data<br>*H. suis*: 0.25-32<br>*H. bizzozeronii*: 0.5-2<br>*H. felis*: 0.5-4<br>*H. salomonis*: 2	Van den Bulck *et al.*, 2005<br>Berlamont *et al.*, 2019
**Neomycin**	2 – 32	0.5 - 2	*H. pylori*: no data<br>*H. suis*: 2-128<br>*H. bizzozeronii*: 0.25-0.5<br>*H. felis*: 0.125-0.5<br>*H. salomonis*: 0.25-0.5	Van den Bulck *et al.*, 2005<br>Berlamont *et al.*, 2019
**Oxytetracycline**	≤0.03125 – 0.25	≤0.03125 - 0.125	*H. pylori*: 0.125-1<br>*H. suis*: no data<br>*H. bizzozeronii*: 0.25-1<br>*H. felis*: 0.125-1<br>*H. salomonis*: 0.125-0.5	Loo *et al.*, 1997<br>Van den Bulck *et al.*, 2005

**Doxycycline**	0.0625 – 0.25	0.0625 - 0.25	*H. pylori*: no data<br>*H. suis*: 0.06-32<br>*H. bizzozeronii*: no data<br>*H. felis*: no data<br>*H. salomonis*: no data	Berlamont *et al.*, 2019
**Metronidazole**	0.25 - 4	0.025 - 0.5	*H. pylori*: 64-256<br>*H. suis*: 0.5-64<br>*H. bizzozeronii*: 1-8<br>*H. felis*: 0.5-16<br>*H. salomonis*: 0.5	Loo *et al.*, 1997<br>Van den Bulck *et al.*, 2005<br>Berlamont *et al.*, 2019
